# *Drosophila* Sex Peptide controls the assembly of lipid microcarriers in seminal fluid

**DOI:** 10.1073/pnas.2019622118

**Published:** 2021-01-25

**Authors:** S. Mark Wainwright, Ben R. Hopkins, Cláudia C. Mendes, Aashika Sekar, Benjamin Kroeger, Josephine E. E. U. Hellberg, Shih-Jung Fan, Abigail Pavey, Pauline P. Marie, Aaron Leiblich, Irem Sepil, Philip D. Charles, Marie L. Thézénas, Roman Fischer, Benedikt M. Kessler, Carina Gandy, Laura Corrigan, Rachel Patel, Stuart Wigby, John F. Morris, Deborah C. I. Goberdhan, Clive Wilson

**Affiliations:** ^a^Department of Physiology, Anatomy and Genetics, University of Oxford, OX1 3QX Oxford, United Kingdom;; ^b^Department of Zoology, University of Oxford, OX1 3PS Oxford, United Kingdom;; ^c^Department of Evolution and Ecology, University of California, Davis, CA 95616;; ^d^Target Discovery Institute Mass Spectrometry Laboratory, Target Discovery Institute, Nuffield Department of Medicine, University of Oxford, OX3 7BN Oxford, United Kingdom;; ^e^Applied Zoology, Faculty of Biology, Technische Universität Dresden, Dresden D-01069, Germany;; ^f^Institute of Infection, Veterinary and Ecological Sciences, University of Liverpool, L69 7ZB Liverpool, United Kingdom

**Keywords:** reproduction, secretion, seminal proteins, triacylglycerides, Sex Peptide

## Abstract

Seminal fluid plays an essential role in promoting male reproductive success and modulating female physiology and behavior. In the fruit fly, *Drosophila melanogaster*, Sex Peptide (SP) is the best-characterized protein mediator of these effects. It is secreted from the paired male accessory glands (AGs), which, like the mammalian prostate and seminal vesicles, generate most of the seminal fluid contents. After mating, SP binds to spermatozoa and is retained in the female sperm storage organs. It is gradually released by proteolytic cleavage and induces several long-term postmating responses, including increased ovulation, elevated feeding, and reduced receptivity to remating, primarily signaling through the SP receptor (SPR). Here, we demonstrate a previously unsuspected SPR-independent function for SP. We show that, in the AG lumen, SP and secreted proteins with membrane-binding anchors are carried on abundant, large neutral lipid-containing microcarriers, also found in other SP-expressing *Drosophila* species. These microcarriers are transferred to females during mating where they rapidly disassemble. Remarkably, SP is a key microcarrier assembly and disassembly factor. Its absence leads to major changes in the seminal proteome transferred to females upon mating. Males expressing nonfunctional SP mutant proteins that affect SP’s binding to and release from sperm in females also do not produce normal microcarriers, suggesting that this male-specific defect contributes to the resulting widespread abnormalities in ejaculate function. Our data therefore reveal a role for SP in formation of seminal macromolecular assemblies, which may explain the presence of SP in *Drosophila* species that lack the signaling functions seen in *D*. *melanogaster*.

## Significance

Seminal fluid plays a critical role in reprogramming female physiology and behavior to promote male reproductive success. We show, in the fruit fly, that specific seminal proteins, including the archetypal “female-reprogramming” molecule Sex Peptide, are stored in male seminal secretions in association with large neutral lipid-containing microcarriers, which rapidly disperse in females. Related structures are also observed in other Sex Peptide-expressing *Drosophila* species. Males lacking Sex Peptide have structurally defective microcarriers and exhibit abnormal transfer of many seminal proteins to females. Our data reveal that this key signaling molecule in *Drosophila* seminal fluid is also a microcarrier assembly factor that modulates transfer of other seminal factors and that this may be a more evolutionarily ancient role of this protein.

In addition to spermatozoa, semen contains a complex mixture of macromolecules and nutrients secreted by the accessory glands of the male reproductive tract. In humans, seminal plasma nutrients include fructose from the seminal vesicles and triglycerides, both major energy sources for sperm in the female (1). In addition, enzymes, such as proteases and lipases, nonenzymatic binding proteins, like lectins and cysteine-rich secretory proteins (CRISPs), and a wide range of hormones and signaling molecules are major components, many of them generated in the prostate gland (2, 3). These molecules may be stored for days in the gland following cellular secretion, prior to being delivered to females during mating, when mixing of seminal plasma components can trigger enzyme and signal activation (4). However, the mechanisms that underpin these storage and activation events are generally not well understood.

**Fig. 1.**
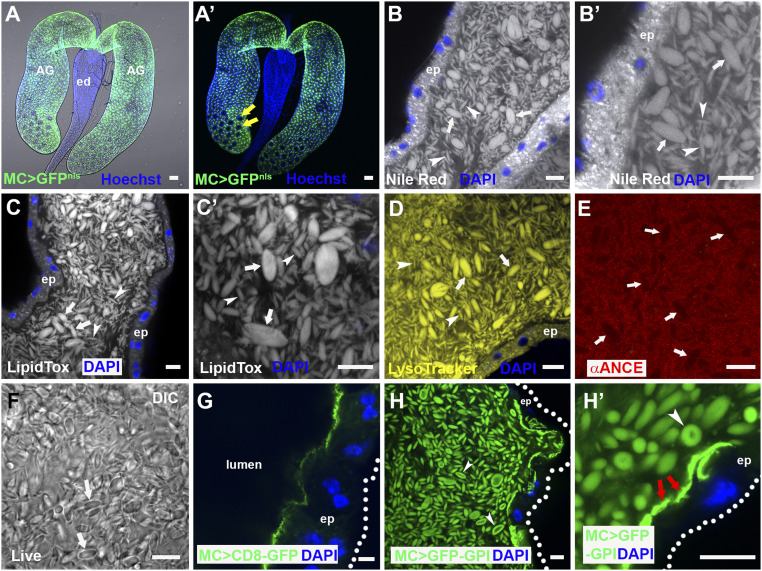
The AG lumen contains abundant lipophilic microcarriers. (*A* and *A*′) Fluorescence image with (*A*) and without (*A*′) bright-field illumination of paired *D. melanogaster* male AGs connecting to the ejaculatory duct (ed). Main cells express nuclear GFP under *Acp26Aa*-GAL4 main cell-specific control (green), but secondary cells in distal tip (two of which are marked by yellow arrows in *A*′) do not. (*B*–*E*) Confocal sections through AG lumen stained with Nile Red (*B*, *B*′; latter is high magnification view), LipidTox (*C* and *C*′), LysoTracker Deep Red (*D*; yellow) and anti-ANCE (red), a soluble secreted protein (*E*). White arrows mark representative large microcarriers, and arrowheads mark small microcarriers. (*F*) DIC image of lumen from living AG also reveals microcarriers (white arrows). (*G*) Transmembrane CD8-GFP expressed in main cells marks the apical plasma membrane, but not luminal microcarriers. (*H* and *H*′) Main cell-expressed GFP-GPI labels microcarriers at their surface (*H* and *H*′, white arrowheads) together with the apical surface of the epithelial monolayer (*H*′, red arrows). Nuclei marked with Hoechst (*A* and *A*′, blue) or DAPI (*B*, *E*, *G*, and *H*, blue). AG epithelium (ep) (dotted white line in *G* and *H* marks approximate basal surface). Main cell-specific *Acp26Aa*-GAL4 driver (MC>). (Scale bars: 10 µm.)

The paired *Drosophila melanogaster* male accessory glands (AGs) share functional similarities with both the prostate and seminal vesicles in humans (5). The monolayer epithelium of these glands is formed from two secretory cell types, about 1,000 main cells and 40 secondary cells at the distal tip (6) (Fig. 1 *A* and *A*′). This glandular epithelial tube surrounds a large lumen. The AG secretome and its functions have been extensively characterized, and multiple bioactive Accessory gland proteins (Acps) identified (7, 8). Several of these induce behavioral and physiological changes in mated females. The archetypal Acp is Sex Peptide (SP or Acp70Aa), a 36-amino acid protein, which is synthesized by main cells (9, 10). On transfer to females following mating, SP effects a comprehensive reprogramming of female physiology and behavior. It promotes long-term increases in egg laying, reduces female receptivity to remating (11, 12), and affects sperm release (13), diet (14), feeding behavior (15), water balance (16), defecation (17), sleep (18), immunity (19), aggression (20), and memory (21).

Maintaining this complex postmating response (PMR) requires SP association with the sperm plasma membrane after mating (12). Sperm can then be stored for several weeks in two female organs, the paired spermathecae and the seminal receptacle, with SP gradually released by proteolytic cleavage to mediate its effects (22).

Studies in which SP or SP mutant peptides are either injected or expressed ectopically in females have demonstrated that SP can induce many of the characterized female PMRs, with distinct molecular domains in SP having different functions (for example, refs. 9 and 23–25). In females, the SP receptor (SPR) is required to mediate most of these effects (26). SPR is expressed in specific neurons of the female reproductive tract that have a key role in the SP-dependent PMR (27, 28), and in other neurons in the central nervous system that are also able to respond to circulating SP (29, 21). In addition, SP appears to produce some SPR-independent PMRs in females (30, 20).

Here, we report an SPR-independent function for SP in males, involving storage and delivery of seminal components. We show that the AG lumen is filled with many large, fusiform- and ellipsoid-shaped microcarriers containing a neutral lipid core and coated with specific proteins, such as SP. Microcarriers rapidly dissipate on transfer to females after mating, providing a simple mechanism for timely release of stored seminal proteins. Surprisingly, we find that SP is essential for assembly of microcarriers in males and that this function is required for the normal delivery of multiple seminal proteins and lipids to the female reproductive tract during mating. Furthermore, we identify related microcarrier structures in other *Drosophila* species that express an SP and show that the size and shape of microcarriers have changed as the amino acid sequence of SP evolved in these species.

## Results

### The Lumen of the AG Is Filled with Large Neutral Lipid-Containing Microcarriers.

**Fig. 2.**
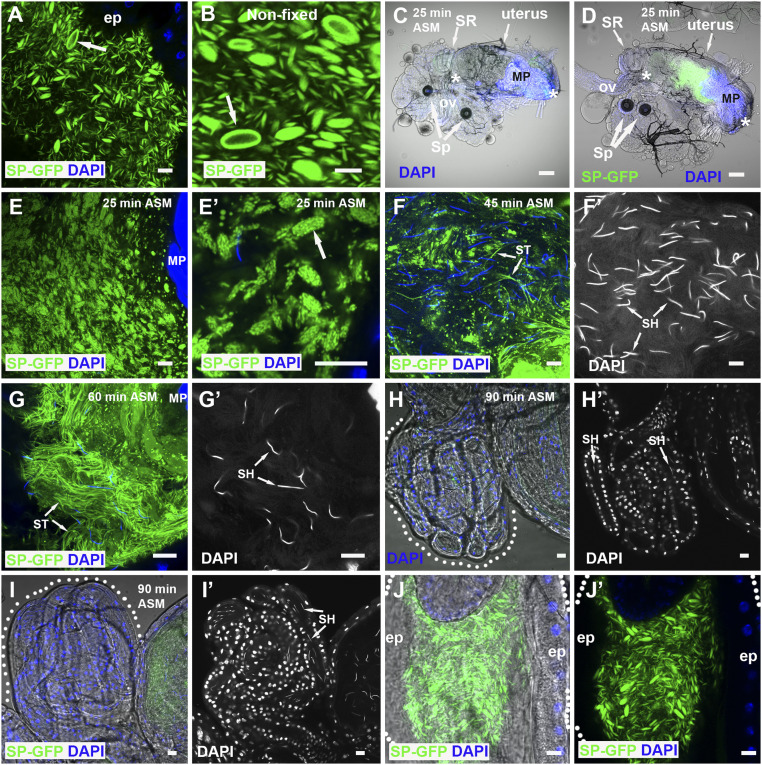
SP-GFP is loaded on microcarriers, which disassemble when transferred to females. (*A* and *B*) SP-GFP (green) marks microcarriers in fixed (*A*) and nonfixed (*B*) AG lumen, coating the surface of the largest structures (arrows). (*C* and *D*) Combined fluorescence and bright-field images of reproductive tract of female mated to a control (*C*) or SP-GFP (*D*) male dissected 25 min ASM. Anterior (*Left*) and posterior (*Right*) limits of uterus are demarcated by white asterisks, and seminal receptacle (SR), paired spermathecae (Sp), common oviduct (ov), and mating plug (MP), which autofluoresces in the DAPI channel, are marked. (*E* and *F*) Higher magnification views of posterior uterus at this time reveal microcarrier structures have changed (*E*) with SP-GFP concentrated in microdomains (*E*′, arrow). (*F* and *G*) Later (45 min ASM), many microcarriers have disassembled, and some SP-GFP has associated with sperm tails (ST, *F* and *F*′) while, later still (60 min ASM), few recognizable microcarriers remain, and many more strongly labeled sperm tails are observed in the anterior uterus (ST, *G* and *G*′). Sperm heads (SH) are marked by DAPI. (*H* and *I*) Labeled sperm tails (ST) are not present in the seminal receptacles (60 to 90 min ASM), which contain sperm heads (SH), both in females mated with control (*H* and *H*′) and SP-GFP males (*I* and *I*′). (*J* and *J*′) Microcarriers remaining in the ejaculatory duct after mating maintain their structure. Outlines of seminal receptacles (*H* and *I*) and ejaculatory duct (*J* and *J*′) are marked by dotted lines. Nuclei marked with DAPI (blue). AG and ejaculatory duct epithelia (ep). (Scale bars: 10 µm, except *C* and *D*, 30 µm.)

While analyzing the lipid content of epithelial cells within the male AG, using the lipophilic dye Nile Red, which stains membranes and lipid droplets, we observed that the large AG lumen is filled with fluorescent fusiform structures typically 3 to 8 µm in length (Fig. 1 *B* and *B*′). These structures were of variable diameter, ranging from less than 0.5 µm to a maximum of 4.0 µm (*SI Appendix*, Fig. S1*F*). Since these structures were found to bind specific main cell proteins (Fig. 2*A*), we call them “microcarriers.” The neutral lipid-specific dye LipidTox Red stained microcarriers highly selectively (Fig. 1 *C* and *C*′), suggesting they contain large quantities of triglycerides and other nonpolar lipids. Microcarriers were also detected using high concentrations of the acidophilic, but partially hydrophobic, LysoTracker dyes (Fig. 1*D*) (31). By contrast, in fixed tissue, microcarriers exclude access to antibodies raised against soluble secreted AG proteins, such as angiotensin I-converting enzyme (ANCE) (Fig. 1*E*). Microcarriers are not an artifact of fixation or staining because they are readily discernible in living glands using differential interference contrast (DIC) microscopy (Fig. 1*F*). In virgin males, they are not observed in other parts of the reproductive tract (e.g., *SI Appendix*, Fig. S1*A*), suggesting they are exclusively made by the AG.

Previous studies have shown that some transmembrane proteins expressed in epithelial secondary cells of the AG are secreted via exosomes (32, 33). When transmembrane proteins were expressed in main cells, they did not associate with microcarriers (Fig. 1*G* and *SI Appendix*, Fig. S1 *B* and *B*′), and neither did dyes like PKH26 that bind to lipid bilayers (*SI Appendix*, Fig. S1 *C* and *C*′). A secreted form of GFP, comprised of the SP signal sequence fused to GFP (25), also failed to preferentially bind to microcarriers (*SI Appendix*, Fig. S1*D*). By contrast, GFP-GPI, a GFP fusion protein carrying the lipid anchor glycosylphosphatidylinositol, strongly labeled microcarriers when expressed in main cells (Fig. 1 *H* and *H*′), but not when made in secondary cells (*SI Appendix*, Fig. S1 *E* and *E*′) (34), consistent with the idea that main cells produce these structures. Indeed, blocking secretion in adult secondary cells by expressing the BMP signaling inhibitor Dad from eclosion onwards (32, 34) had no obvious effect on microcarrier production (*SI Appendix*, Fig. S1 *G* and *H*). However, microcarrier shape was frequently more bimodal than in control glands, with many thread-like microcarriers produced, suggesting that secondary cells may affect the final morphology of these structures. When GFP-GPI was overexpressed in main cells, a concentrated layer of GFP-GPI–positive staining was observed apically (Fig. 1*H*′), reflecting the shedding of lipophilic material from these cells. In the largest microcarriers, GFP-GPI, unlike LipidTox staining (Fig. 1 *C* and *C*′), was surface-localized (Fig. 1 *H* and *H*′), suggesting that these structures have a distinct outer layer, most likely a phospholipid monolayer into which the GPI anchor is inserted, surrounding the neutral lipid core. Although microcarrier ultrastructure was difficult to preserve for transmission electron microscopy, micrographs were consistent with these structures having a homogeneous internal structure (*SI Appendix*, Fig. S1*I*).

### SP Is a Microcarrier Cargo.

An SP-GFP C-terminal GFP fusion protein expressed in main cells under the control of *SP* gene regulatory elements (35) has previously been used to assess SP transfer to females. Surprisingly, we found that, in the presence of wild-type SP, it strongly associates with microcarriers and concentrates at the surface of the largest structures but is present at very low levels within main cells (Fig. 2 *A* and *B* and Movie S1). When these SP-GFP males were mated, fluorescently labeled microcarriers were transferred to females (Fig. 2 *C* and *D*). We were unable to detect microcarriers using neutral lipid stains in the female reproductive tract, at least partly because of poor dye penetration. However, using SP-GFP as a marker, we found that, 25 min after the start of mating (ASM), which is typically within 5 min of the end of mating, microcarriers had already started to change their morphology (Fig. 2 *E* and *E*′). Although their basic fusiform shape was frequently still distinguishable, SP-GFP concentrated in microdomains on the microcarrier surface. Later, at 45 min ASM, smaller spherical SP-GFP–positive puncta were dispersed throughout the uterus, and SP-GFP was observed on a subset of sperm tails (Fig. 2 *F* and *F*′) while later still (60 min ASM), more of the SP-GFP (Fig. 2 *G* and *G*′) was associated with sperm tails. However, at 90 min ASM, only very weak, if any, GFP expression was observed on sperm in the sperm storage organs (Fig. 2 *H* and *I*′), either because the most strongly labeled sperm do not migrate to these organs or because the GFP tag or fluorescence is lost over time in females. Microcarriers that are ejected from the AG, but are trapped in the male ejaculatory duct at the end of mating, do not break down or form SP-GFP—labeled subdomains at their surface (Fig. 2 *J* and *J*′), events exclusively seen in females. This suggests that microcarrier dissipation is only triggered by physical or chemical signals when these structures enter the uterus.

To confirm that C-terminal tagging of SP with GFP does not affect SP’s binding properties in the AG lumen and to begin to dissect out what domains in SP bind to microcarriers, we overexpressed three SP-GFP fusions in main cells under GAL4/ upstream activating sequence (UAS) control: the N-terminal half of mature SP fused at its C terminus to GFP (SPn-GFP), the C-terminal half of SP fused at its N terminus to GFP (GFP-SPc), and a fusion with GFP located in the center of the SP protein (SPn-GFP-SPc). The latter has been shown to have biological activity in females (25). Using the main cell-specific *Acp26Aa*-GAL4 driver (Fig. 1*A*) (11), which expresses at lower levels than GFP-tagged SP under its own promoter, all SP fusions partitioned with microcarriers (*SI Appendix*, Fig. S1 *J*–*L*), albeit less selectively for the N-terminal SP construct, SPn-GFP. Microcarriers therefore appear to bind to both the N- and C-terminal domains of SP. We conclude that, in addition to neutral lipids, they act as stores in males for SP and potentially other associated seminal proteins, such as those with a GPI anchor, and serve as vehicles for their transfer to females. Regulated microcarrier disassembly in females presumably assists in the timely release of lipids and seminal proteins, such as SP, after mating.

### SP Controls Microcarrier Morphology via an SPR-Independent Mechanism.

**Fig. 3.**
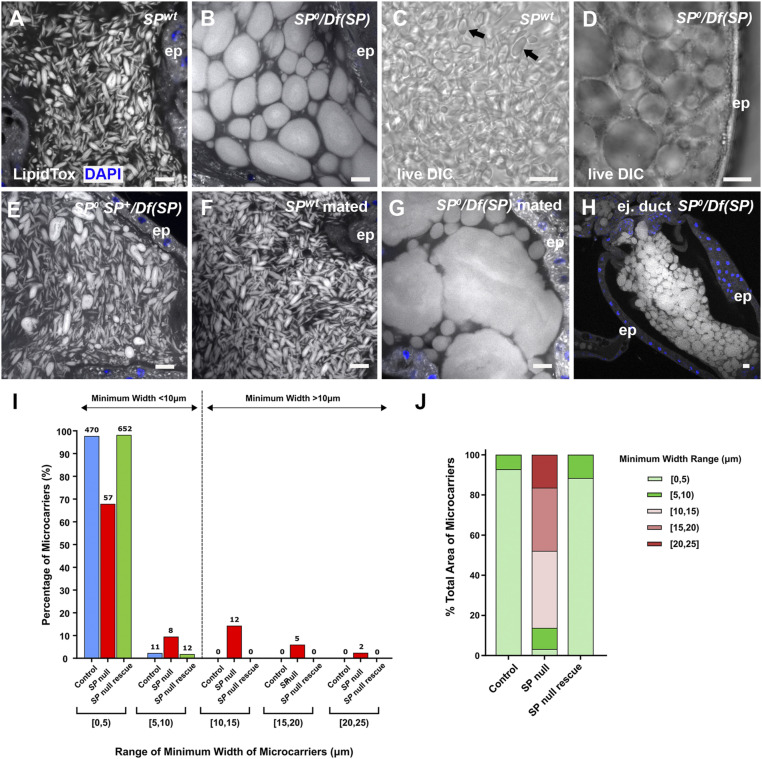
SP is essential for proper assembly of microcarriers. (*A* and *B*) Confocal images of LipidTox-labeled microcarriers in lumen of AG from control (*A*) and *SP*^*0*^*/Df(SP)* null (*B*) males. Mutant male has grossly enlarged microcarriers. (*C* and *D*) DIC images of living AGs dissected from control (C, black arrows) and *SP*^*0*^*/Df(SP)* (*D*) males. (*E*) Microcarrier structural defects in *SP*^*0*^*/Df(SP)* null males are rescued by a genomic *SP* construct *SP*^*0*^ *SP*^*+*^*/Df(SP)*. (*F* and *G*) Microcarriers enlarge further after multiple matings in *SP*^*0*^*/Df(SP)* null (*G*), but not in wild-type (*F*) males. (*H*) In *SP*^*0*^*/Df(SP)* null males, these enlarged microcarriers are observed when seminal fluid remains in the lumen of the ejaculatory duct after mating. (*I* and *J*) Microcarrier size and area profiles for glands shown in A, B, and E. Microcarrier outlines were detected in images of the AG lumen using CellProfiler Software version 2.2.0 (*Materials and Methods*) and then grouped according to minimum width range (*I*) or percentage of luminal area occupied by microcarriers in each width range (*J*). Numbers of microcarriers within each size range are shown above bars (*I*). *SP*^*0*^*/Df(SP)* null glands have considerably fewer small microcarriers (<10 µm) and more large microcarriers (>10 µm) than the other genotypes. The enlarged microcarriers in *SP*^*0*^*/Df(SP)* null glands contain most of the lipid in the AG lumen, as estimated by LipidTox staining area. Nuclei marked with DAPI (blue). AG (*A*–*G*) or ejaculatory duct (*H*) epithelium (ep). (Scale bars: 10 µm.)

To assess whether *SP* is involved in microcarrier assembly, we analyzed AGs of males carrying the previously generated *SP*^*0*^ null allele (12), either as a homozygote or in transheterozygous combination with a small *SP* deficiency, *Df(3L)Δ130* (36, 12). These transheterozygous *SP* null males have been used to characterize the full range of *SP* mutant PMR phenotypes (12–21). Unexpectedly, these mutant animals displayed dramatic defects in microcarrier morphology (Fig. 3 *A*–*D*, *I*, and *J* and *SI Appendix*, Figs. S2 *A* and *B* and S3*A*). Most microcarrier-like structures were highly enlarged and either spherical or ellipsoid in shape. The enlarged microcarrier phenotype was never observed in wild-type glands (Fig. 3 *A* and *C*). In confocal images of the AG lumen, 10 of 10 *SP*^*0*^*/Df(SP)* null glands had microcarriers with a minimum width greater than 10 µm whereas 0 of 10 wild-type glands contained such structures (*P* < 0.0001; Fisher’s exact test). The enlarged microcarriers from the *SP*^*0*^*/Df(SP)* null glands were uniformly stained by LipidTox. They appeared like large lipid droplets under DIC (Fig. 3*D*). The defects were absent in *SP*^*0*^ *SP*^*+*^*/Df(3L)Δ130* males, which express an *SP* genomic rescue construct that rescues the PMR phenotypes in mated females (12) (Fig. 3*E*); 0 of 10 *SP* rescue glands had microcarriers with a minimum width greater than 10 µm [*P* < 0.0001 versus *SP*^*0*^*/Df(SP)*]. Automated measurement of minimum microcarrier diameter in individual images of male AGs with these different genotypes confirmed the change in size distribution in the *SP* null background (Fig. 3*I*). Mating *SP*^*0*^*/Df(SP)* null males multiple times with females over several days to mix and eject the AG’s contents exacerbated the mutant phenotype, with some microcarriers spanning the entire diameter of the AG lumen (Fig. 3 *F* and *G*), suggesting that microcarriers can enlarge by fusion. When seminal fluid remained in the ejaculatory duct of *SP*^*0*^*/Df(SP)* null males after mating, the duct lumen was also filled with enlarged microcarriers (Fig. 3*H*).

**Fig. 4.**
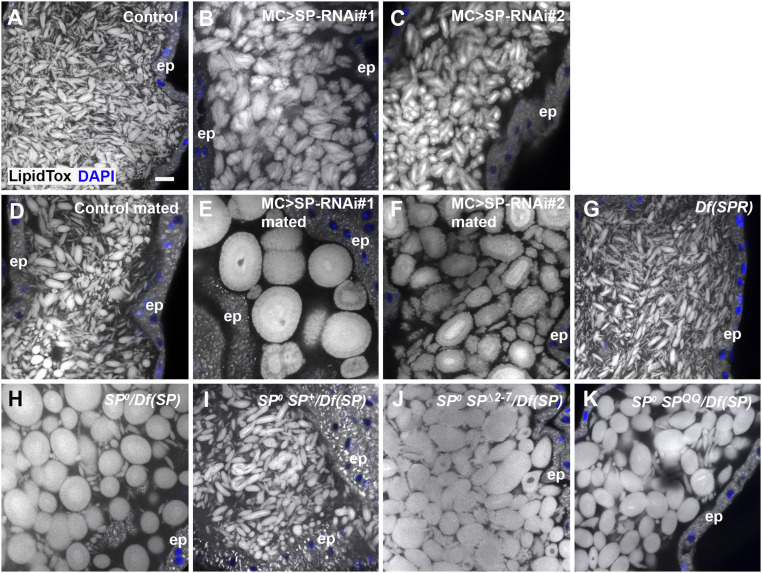
Knockdown of *SP* in main cells also produces highly enlarged microcarriers. All specimens are stained with LipidTox. (*A*) Confocal image of microcarriers in lumen of AG from control male. (*B* and *C*) Knockdown of *SP* in main cells at 29 °C with two RNAis, *UAS-SP-RNAi#1* (*B*; IR2 from ref. 11) and *UAS-SP-RNAi#2* (*C*; TRiP.JF02022) produces enlarged microcarriers. (*D*–*F*) Multiple mating of *SP* knockdown males leads to further increases in microcarrier size (*E* and *F*), presumably via fusion, which is not observed in controls (*D*). (*G*) *SPR* mutant males [homozygous *Df(1)Exel6234*] have normal microcarriers. (*H*–*K*) The *SP*^*0*^*/Df(SP)* null phenotype (*H*) is rescued by a wild-type *SP* genomic construct in *SP*^*0*^ *SP*^*+*^*/Df(SP)* males (*I*), but not by genomic constructs expressing mutant SP^∆2-7^ (*J*) or SP^QQ^ (*K*). Nuclei marked with DAPI (blue). AG epithelium (ep). (Scale bar: *A*, 10 µm, applies to all panels.)

To confirm that SP expression in main cells is required for normal microcarrier assembly, we knocked down *SP* transcripts specifically in these cells, using the GAL4/UAS system (37), employing the *Acp26Aa*-GAL4 driver (11). Although limited effects were observed when these experiments were performed at 25 °C, expression of *SP-*RNA interference (RNAi) constructs from three different transgenic lines at 29 °C, a temperature that typically enhances GAL4-induced expression (38), produced consistent marked defects in microcarrier morphology (Fig. 4 *A*–*C* and *SI Appendix*, Figs. S2 *C* and *D* and S3*B*). Microcarriers were enlarged in all three knockdowns, although to a lesser extent than in *SP*^*0*^*/Df(SP)* null males. As observed in mated *SP*^*0*^*/Df(SP)* null males (Fig. 3*G*), mating greatly exacerbated the size phenotype (Fig. 4 *E* and *F*).

In females, many of SP’s activities in modulating the female PMR are mediated by the SPR (26). However, *SPR* mutant males displayed completely normal microcarriers (Fig. 4*G*), demonstrating that SP acts independently of the SPR in the male AG, presumably via direct interaction with microcarriers.

Binding of SP to the plasma membrane of sperm in females requires a short peptide sequence at the N-terminal end of the mature molecule (22). The C-terminal region of SP is proteolytically detached from sperm in the sperm storage organs over a 2-wk period so that it can interact with SPR and other potential targets. Two mutants expressed under the control of the SP promoter, one that lacks the N-terminal membrane-association domain (SP^∆2-7^) and the other mutated at the proteolytic cleavage site (SP^QQ^), have both previously been shown to fail to induce the long-term PMR in females (22). These constructs also failed to rescue the microcarrier phenotype in *SP*^*0*^*/Df(SP)* null males (Fig. 4 *H*–*K* and *SI Appendix*, Fig. S3*C*). For both mutants, seven of seven glands had microcarriers with a minimum width greater than 10 µm (*SI Appendix*, Fig. S3*C*), suggesting that the N-terminal region of SP, which appears to bind to microcarriers (*SI Appendix*, Fig. S1*F*), plays an important role in microcarrier assembly, as well as sperm binding.

### Microcarriers from *SP* Mutant Males Do Not Disassemble Normally in Females after Mating.

**Fig. 5.**
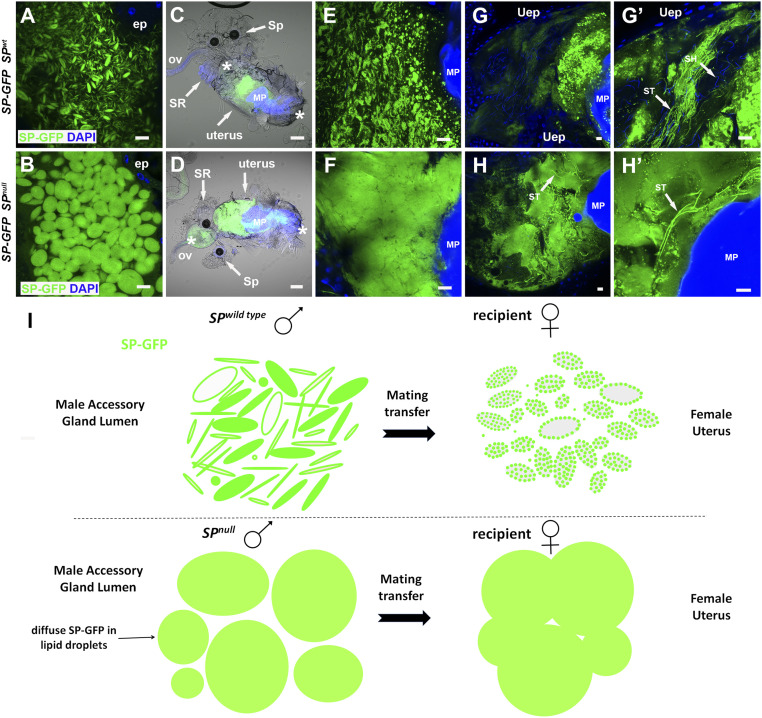
Microcarriers from *SP* null males do not dissipate normally when transferred to females during mating. (*A* and *B*) A genomic SP-GFP fusion construct labels *SP* wild-type microcarriers (*A*), and enlarged defective microcarriers in the AG of *SP*^*0*^*/Df(SP)* null males although it does not rescue the associated microcarrier phenotype (*B*). (*C*–*F*) Combined fluorescence and bright-field images at 25 to 30 min ASM of whole reproductive tracts (anterior on left; *C* and *D*) and posterior uterus at higher magnification (*E* and *F*) from females mated either with control males expressing SP-GFP (*C* and *E*) or with *SP*^*0*^*/Df(SP)* null males expressing SP-GFP (*D* and *F*). Microcarrier-like structures from the *SP*^*0*^*/Df(SP)* null male are fused together in a globular mass whereas microcarriers from control males do not fuse but carry localized SP-GFP puncta. (*G* and *H*) At 45 to 50 min ASM, SP-GFP–positive material remains in a globular mass in females mated with *SP*^*0*^*/Df(SP)* null males, which extends into the anterior uterus, unlike controls (*H* and *H*′). This mass contains a few intensely labeled sperm tails (arrows). By contrast, SP-GFP from wild-type males has dispersed although some intense fluorescent puncta remain (*G* and *G*′), and often many sperm tails in the anterior uterus are labeled (arrows in *G*′). (*I*) Schematic representing microcarrier structure in AGs of wild-type and *SP*^*0*^*/Df(SP)* null males, as visualized using the SP-GFP fusion protein, and the changes that take place 25 to 30 min ASM in the female reproductive tract. In *C* and *D*, anterior (left) and posterior (right) boundaries of uterus are demarcated by asterisks, and seminal receptacle (SR), one of the two spermathecae (Sp), oviduct (ov), and mating plug (MP) are labeled. Nuclei marked with DAPI (blue). AG epithelium (ep), uterine epithelium (Uep). (Scale bars: 10 µm, except for *C* and *D*, 30 µm.)

A key property of microcarriers is that they are stable in the male AG yet change their morphology within minutes, when transferred to females. We tested how this process is affected in *SP* mutants. Since the C-terminally tagged SP-GFP construct, which has previously been reported to lack normal SP activity in females (23), failed to rescue the *SP*^*0*^*/Df(SP)* null microcarrier phenotype in males (Fig. 5*B*), we used this as an alternative to neutral lipid dyes to mark microcarriers. SP-GFP distributed evenly throughout the enlarged microcarriers in *SP*^*0*^*/Df(SP)* null males (Fig. 5 *A* and *B*).

Unlike in controls (Fig. 5*C*), microcarriers from *SP*^*0*^*/Df(SP)* null males failed to rapidly dissipate in females and instead formed a homogeneously stained mass in the uterus (Fig. 5*D*), which did not break down during the period when SP-GFP is normally transferred to sperm tails (compare Fig. 5 *G* and *G*′ with Fig. 5 *H* and *H*′); indeed, unlike controls, the mass extended into the anterior uterus with some sperm tails embedded within it. We conclude that normal dissipation and distribution of microcarrier cargos is disrupted in females mated with *SP*^*0*^*/Df(SP)* null males (Fig. 5*I*), and this is likely to contribute to the aberrant postmating phenotypes observed in mated females.

### Loss of SP Remodels the Seminal Proteome.

To assess the effect of *SP* loss of function on the transferred seminal proteome, we compared the AG proteome of 4- to 5-d-old virgin and mated males, with either the null *SP*^*0*^*/Df(3L)Δ130* or *SP*^*0*^ *SP*^*+*^*/Df(3L)Δ130* rescue genotype. Focusing on the 116 detected known seminal fluid proteins (SFPs), which are generally reduced in relative levels following mating, a principal component analysis (PCA) biplot showed separation of samples according to both mating (PC1) and genotype (PC3), suggesting that loss of *SP* leads to compositional changes in the seminal proteome (*SI Appendix*, Fig. S4*A*). A hierarchical clustering analysis identified distinct profiles of SFP change across matings and genotypes that contribute to modulation of composition. By applying this analysis across glands, we could test for generalized behaviors of groups of SFPs in terms of production, postmating retention, and the difference between these, which represents what is transferred to females (39).

**Fig. 6.**
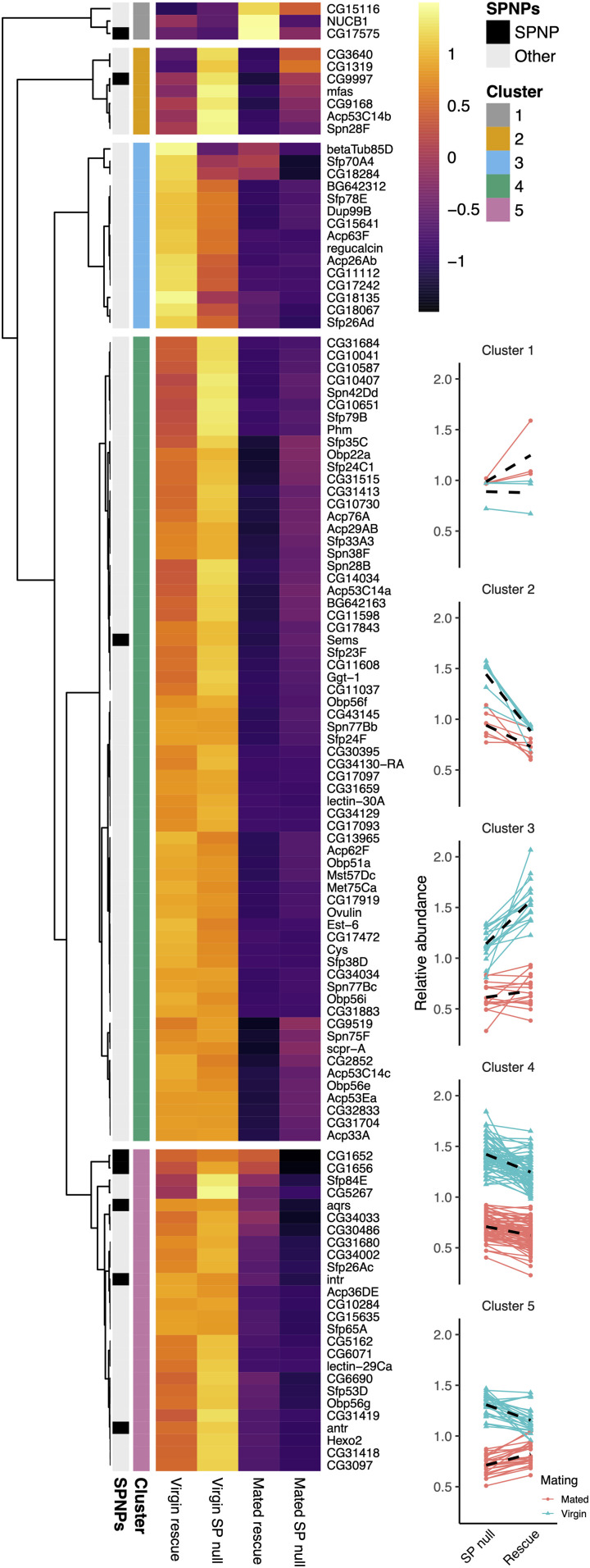
*SP* loss differentially modifies the transfer of specific subclasses of seminal protein. Heat map shows mean log_2_ abundance taken across three replicates for each SFP. Each protein is plotted for the rescue control virgin (far left) and mated (middle right column), and *SP* null virgin (middle left) and mated (far right) glands. SP network proteins (SPNPs) are marked (black on the left), in addition to the five clusters. Mean-centered abundance patterns for each protein in clusters 1 to 5 are shown on *Right*. Red, mated glands; blue, virgin glands. Black dashed lines give the average response for a mating treatment.

Hierarchical clustering revealed the presence of five distinct, higher order clusters (Fig. 6). We ran linear mixed effects models on each to test for associations with our measured variables. Clusters 3 and 5 showed significant associations with the interaction between genotype and mating (cluster 3: F_1,42_ = 4.736, *P* = 0.035; cluster 5: F_1,75_ = 24.564, *P* < 0.0001). The average response of SFPs in cluster 3 was for reduced transfer to females in *SP* nulls. Cluster 3 includes Dup99B, a protein expressed in the anterior ejaculatory duct, which is consistently detected in AG preparations and shares considerable structural and functional similarity with SP, particularly in its C-terminal region (24). Furthermore, analysis of proteins not classed as SFPs with a genotype/mating interaction (*SI Appendix*, Fig. S4*B*) identified the GPI-anchored protein Contactin within a group of eight proteins that appear to be selectively retained in the glands of *SP* null males after mating (*SI Appendix*, Fig. S4*B*, cluster 1). Therefore, at least two proteins that might be expected to bind to microcarriers appear to be preferentially retained in *SP* null males where these structures fail to form normally.

By contrast, SFPs in cluster 5 were transferred to females in greater quantities in *SP* null males (Fig. 6). This elevated transfer is driven by two processes: greater production in virgins and lowered postmating retention. Interestingly, despite accounting for just 26 of the 116 SFPs, cluster 5 was significantly enriched for SP network proteins (*P* = 0.014; Fisher’s exact test), containing five of the eight known: CG1652, CG1656, Antr, Intr, and Aqrs (40–43), suggesting an association between these proteins, SP, and microcarriers.

Clusters 2 and 4 did not show a significant interaction term between genotype and mating (cluster 2: F_1,18_ = 3.103, *P* = 0.095; cluster 4: F_1,192_ = 2.873, *P* = 0.092) but did show significant associations with genotype (cluster 2: F_1,19_ = 44.042, *P* < 0.0001; cluster 4: F_1,193_ = 40.401, *P* < 0.0001). In both clusters, the average response was for SFPs to be at higher abundance in *SP* null glands, consistent with elevated production in the absence of *SP*. Cluster 1, which contained only three SFPs, showed no significant associations with genotype (G × M: F_1,6_ = 44.042, *P* = 0.949; G: F_1,7_ = 0.579).

In summary, the transfer of at least two subclasses of SFPs in the seminal proteome is significantly modulated in an *SP* null background while many other proteins are transferred normally, despite some being produced in higher quantities. This is consistent with our hypothesis that SP influences the seminal proteome in males, presumably via its role in microcarrier assembly.

### SP and Microcarrier Structure Have Rapidly Coevolved in *Drosophila* Species.

**Fig. 7.**
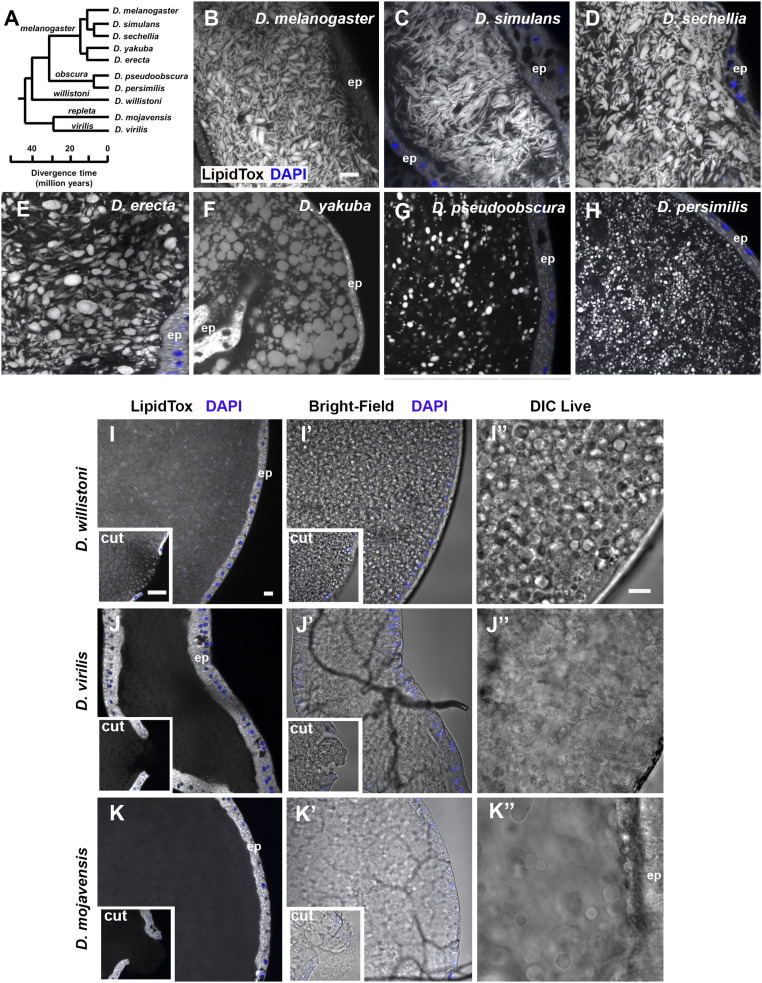
Coevolution of microcarrier morphology and SP in *Drosophila* species. (*A*) Phylogenetic tree of *Drosophila* species used in this study. All species except *D. mojavensis* have a putative SP homolog. Adapted from flybase.org/blast/. (*B*–*K*) LipidTox staining of AGs from 6-d-old virgin males from selected *Drosophila* species, namely *D. melanogaster* (*B*), *D. simulans* (*C*), *D. sechellia* (*D*), *D. erecta* (*E*), *D. yakuba* (*F*), *D. pseudoobscura* (*G*), *D. persimilis* (*H*), *D. willistoni* (*I*), *D. virilis* (*J*), and *D. mojavensis* (*K*). For (*I*–*K*), where LipidTox microcarriers are not readily detectable, bright-field images of the same glands are shown (*I*′–*K*′), as well as DIC images (*I*′′–*K*′′) of different glands. *Insets* in *I–K*) are images of AG with epithelial layer punctured to fully expose luminal contents to LipidTox stain, revealing stained structures only in *D. willistoni*. Note that different subgroups have noticeably different microcarrier size, shape, and density. Nuclei marked with DAPI (blue). AG epithelium (ep). (All scale bars: 10 µm; scale bar in *B* applies to *B*–*H*; scale bar in *I*, applies to *I*–*K* and *I*′–*K*′; and scale bar in *I*′′ applies to *I*′′–*K*′′.)

To test whether other species within the genus *Drosophila* might employ a similar neutral lipid-based strategy to package molecules in seminal fluid, we stained the AGs of multiple *Drosophila* species with LipidTox (Fig. 7). Species closely related to *D. melanogaster*, namely *Drosophila simulans* and *Drosophila sechellia* (Fig. 7*A*), had microcarriers with remarkably similar size and shape (Fig. 7 *B*–*D*). AGs of the species *Drosophila erecta* and *Drosophila yakuba*, which are still members of the *melanogaster* group but have more divergent SP structure (25) (*SI Appendix*, Fig. S5), also contained microcarriers, but these were more spherical in shape (Fig. 7 *E* and *F*).

Examining more distantly related *Drosophila* species with more diverged SP proteins (*SI Appendix*, Fig. S5) (25) revealed very different microcarrier organization. *Drosophila pseudoobscura* and *Drosophila persimilis*, both members of the *obscura* group, have smaller spherical microcarriers that appear to be more widely separated (Fig. 7 *G* and *H*).

Two further *Drosophila* species, *Drosophila willistoni* and *Drosophila virilis*, express forms of SP with major structural differences to the *melanogaster* and *obscura* groups (25). These proteins not only lack a central 10-amino acid portion of the molecule, including the sequence required for proteolytic cleavage, but have also diverged considerably in other regions, with the exception of the last 15 C-terminal amino acids (*SI Appendix*, Fig. S5). Bright-field and DIC microscopy revealed *D. willistoni* glands have densely packed globular structures that can be stained with LipidTox although this is most clearly observed in punctured glands (Fig. 7 *I*–*I′′*). The *D. virilis* AG, whether intact or cut, showed little evidence of LipidTox-stained microcarriers (Fig. 7*J*), and bright-field and DIC imaging suggested a more uniform “flocculence” (Fig. 7 *J*′ and J′′). Finally, we examined the AGs of *Drosophila mojavensis*, a species that lacks an SP homolog (*SI Appendix*, Fig. S5) (25). Although there were more dispersed large spherical structures in the gland lumen in DIC images, no LipidTox-positive staining was observed in these glands (Fig. 7 *K* and *K*′). Staining AGs of these different *Drosophila* species with LysoTracker Deep Red revealed an identical pattern of microcarrier presence, shape, and size (*SI Appendix*, Fig. S6), indicating that the lack of microcarrier staining with LipidTox in *D. virilis* and *D. mojavensis* is not a dye-specific phenomenon. Therefore, our analysis suggests that the evolutionary divergence of SP structure closely parallels changes in microcarrier shape, size, and density.

## Discussion

Seminal fluid plays an essential role in male reproductive success. In *D. melanogaster*, SP, produced from the male AG, has been highlighted as a central player in this process, acting via receptors in the female to stimulate changes that increase fecundity and prevent remating. Here, we demonstrate that SP has an additional, unsuspected role in males in the assembly of neutral lipid-containing microcarriers in the AG lumen (summarized in Fig. 5*I*). These microcarriers store SP and can carry other proteins with lipid anchors. Furthermore, our proteomics analysis reveals that the normal delivery of subgroups of SFPs to females during mating requires SP, potentially because these subgroups interact differently with microcarriers. Microcarrier interactions are likely to also affect dispersal of these proteins in the female reproductive tract. Our analysis of microcarriers in other *Drosophila* species reveals that SP’s microcarrier assembly function may exist in species in which SP has more limited roles in modulating the PMR, suggesting that the former function might have been critical in the evolution of this molecule.

### Lipid Microcarriers Provide a Store, Delivery Vehicle, and Dispersal Machinery for a Subset of Seminal Proteins.

Seminal proteins are produced throughout adult life, but these proteins are only transferred to females sporadically. Some of these proteins are then rapidly activated via mechanisms that are thought to include proteolytic cleavage and pH changes in the female reproductive tract (discussed in ref. 5). Our data suggest that microcarriers could contribute to this activation process. They are repositories for main cell-derived seminal proteins, which presumably partition from the aqueous phase of the AG’s secretions, either because of their lipophilicity or because they have binding partners on the microcarrier surface. In the male, molecules like SP bind specifically to microcarriers and not to AG epithelial cells, strongly suggesting that these surfaces are structurally distinct. Subsequent microcarrier dissipation in the female reproductive tract provides a mechanism for dispersing proteins like SP so they can associate with receptors and cell membranes following mating.

Although both staining of normal microcarriers with lipophilic dyes and the homogeneous internal structure of large defective *SP*^*0*^*/Df(SP)* null “microcarriers” observed with DIC strongly suggest that neutral lipids are a major component of these structures, their precise composition remains unclear. In addition, their nonspherical shape in wild-type males suggests that architectural proteins are highly likely to be involved in establishing their final structure, a proposal supported by the *SP* mutant phenotype. It will now be important to identify these other structural constituents and to establish whether any of these, unlike SP, play evolutionarily conserved roles in seminal fluid production outside the *Drosophila* genus.

Analysis of transcriptomics data from adult *Drosophila* organs reveals high level expression in the AG of multiple lipases that are predicted to be secreted (e.g., CG5162, CG11598, CG11600, CG11608, CG13034, CG18258, CG18284, CG31872, and CG34447) (44, 45), with all having been detected in proteomics analyses of seminal fluid (8, 46). These include proteins sharing homology with triacylglycerol lipases (e.g., CG5162, CG13034, CG18258, and CG34447). These lipases provide a potential mechanism to break down neutral lipid transferred in microcarriers to females so the products can be used as fuel. Mammalian seminal fluid also contains lipases (47–49) and triacylglycerides (50, 51), suggesting that the latter may be required, perhaps as a male-derived nutrient source, in the reproductive system of all higher organisms.

Our identification of extracellular neutral lipid microcarriers as accessible stores of specific seminal proteins is reminiscent of the role of intracellular lipid droplets in storage of cytoplasmic and nuclear proteins (52, 53). Lipid droplets are able to dock with specific intracellular organelles to mediate their functions and deliver their cargos. It will be interesting to investigate whether the remnants of microcarriers, such as the microdomains observed with SP-GFP, are in any way targeted to specific cells or structures after transfer to females as these storage vehicles break down.

It has previously been reported in *Drosophila* that males can adaptively modulate the relative balance of seminal proteins, including SP, in the ejaculate, depending on female mating status and the presence of rival males (54, 55). Loading of selected proteins onto microcarriers might provide a simple mechanism to control such rapid changes if the transfer of these large structures can be differentially regulated compared to soluble proteins: for example, by controlling the opening of the sphincters through which seminal fluid passes from the AGs to the ejaculatory duct.

### Regulation of Microcarrier Morphology by SP and Microcarrier/SP Coevolution in *Drosophila*.

Our study reveals a previously unsuspected male-specific, SPR-independent role for SP in regulating microcarrier shape and size. *SP* mutants in *D. melanogaster* still have neutral lipid-containing structures, but they appear to aggregate and fuse, particularly after mating, to generate large lipid droplet-like structures that no longer retain molecules like SP at their surface. To date, we have not been able to separate the different activities of SP in males and females through expression of different mutants or altered SP levels, making it difficult to fully gauge the importance of the male-specific microcarrier function. However, the observation that *SP* mutants, which were known to affect binding of SP to the surface of sperm or its subsequent release, also fail to rescue the microcarrier defect in *SP* null males suggests that the interpretation of the phenotypes associated with these mutants requires some reevaluation.

Our data suggest that both the C-terminal and N-terminal domains of SP can interact with microcarriers even though they share no structural similarity. This may involve direct binding to the outer surface of the microcarrier or, because both domains contain charged residues, more indirect associations via other molecules attached to microcarriers. The multidomain interaction contrasts with sperm binding and may underlie why SP can transfer to sperm in the female reproductive tract.

Tsuda et al. (25) have suggested that SP is likely to have roles in addition to its effects mediated via SPR signaling in the female reproductive tract, which include induction of a female sexual refractory period. This is because some SP-expressing species, like *D. pseudoobscura* and *D. persimilis*, do not appear to express SPR in this location and additionally show much less female postmating refractoriness relative to other SP-producing species (56). Our data (Fig. 6) suggest that microcarrier assembly may be this additional function, with the shape of microcarriers rapidly coevolving with SP. An absence of microcarriers in species with a highly divergent (*D. virilis*) or no (*D. mojavensis*) SP homolog, as evidenced by two different staining methods, supports our hypothesis. Not unexpectedly, DIC microscopy suggested that the luminal content of these latter two species is not homogenous, but it is clearly different from the other *Drosophila* species we studied (Figs. 1*F* and 7 *I′′*–*K′′*). Interestingly, *D. virilis* expresses SPR in the female reproductive tract so, unlike in other species, its SP protein may be specifically involved in activating the female PMR, rather than microcarrier formation.

In light of these findings, it will now be important to investigate whether other proteins with fundamental roles in packaging and storing seminal fluid components have also evolved signaling roles in animals.

### SP Modulates the Composition of the Seminal Proteome.

An important conclusion from our study is that the normal transfer of different subgroups of SFPs is dependent on SP. One simple explanation is that this reflects differences in their interactions with microcarriers. Having shown that main cell-expressed GFP-GPI binds to microcarriers, it was interesting to identify the GPI-anchored junctional protein Contactin as one of the proteins, which appears to be retained more in the AGs of *SP* null males. Furthermore, preferential retention of Dup99B in *SP* null males is consistent with the idea that this SP-like protein might bind to microcarriers even though it is primarily expressed in the adjacent ejaculatory duct epithelium.

Elegant studies by Wolfner and coworkers have identified several long-term response (LTR) network genes expressed in the AG that are interdependent and required either in the male or female for SP to be retained in the sperm storage organs (40–43). We noticed that several of these proteins appear to be expressed at higher levels in *SP* nulls and also that a greater proportion is transferred from mutant males to females upon mating. A previous study has suggested that two of these proteins, CG1652 and CG1656, are present at similar levels in the female reproductive tract 1 h ASM to *SP* null and *SP* rescue males (40). We cannot easily explain this difference, but it is important to emphasize that our study measures the relative quantity of these proteins that leaves the male AG, not what remains in the female reproductive tract some time later. Overall, our proteomics analysis clearly shows that SP modulates the transfer of specific subclasses of SFPs to females, and, particularly in the case of proteins that are retained in *SP* nulls, this could result from the disruption of microcarriers.

It will now be important to investigate whether any of the network genes is involved in loading or unloading SP from microcarriers or, indeed, whether they play a role in microcarrier assembly, particularly since they appear to be present in species where SP does not seem to be involved in signaling (57). The role of secondary cells in microcarrier morphology also needs to be examined in more detail. Furthermore, confirming that other SFPs identified in the proteomics analysis or main cell-expressed GPI-anchored proteins are microcarrier cargos should allow the functions of these structures to be assessed more extensively and may suggest molecular tools that could be used to screen for similar processes in higher organisms.

## Materials and Methods

### *Drosophila* Stocks and Genetics.

Fly stocks were obtained from the following sources: The Bloomington *Drosophila* Stock Center provided *UAS-GFP.nls* (58), *UAS-mCD8-GFP* (59), *tub-GAL80*^*ts*^ (60), *UAS-SP-RNAi#2* TRiP.JF02022 (61), and *UAS-mCD8-ChRFP*; the Vienna *Drosophila* Resource Center provided *UAS-SP-RNAi#3* (v109175); the Kyoto *Drosophila* Genetic Resource Consortium (DGRC) Stock Center provided *spi-GAL4* (62); S. Goodwin, University of Oxford, Oxford, UK, provided *dsx-GAL4* (63), *Acp26Aa-GAL4* (11), and *SP-GFP* (35); T. Aigaki, Tokyo Metropolitan University, Tokyo, Japan, provided *UAS-SPn-GFP-SPc, UAS-SPn-GFP, UAS-GFP-SPc,* and *UAS-sGFP* (25); M. Wolfner, Cornell University, Ithaca, NY, provided *SP*^*QQ*^*, SP*^*∆2-7*^ (22), and *Df(SPR)* (25); S. Eaton, Max Planck Institute of Molecular Cell Biology and Genetics, Dresden, Germany, provided *UAS-GFP-GPI* (64); T. Chapman, University of East Anglia, Norwich, UK, provided *SP*^*0*^*, SP*^*0*^ *SP*^*+*^ (12), *Df(3L)Δ130* (36), and *UAS-SP-RNAi-IR2* (RNAi#1 (11)); B. Edgar, University of Utah, Salt Lake City, UT, provided esg^ts^F/O (*w; esg-GAL4, UAS-GFPnls; act > CD2 > GAL4, UAS-FLP*); D. Bennett, University of Liverpool, Liverpool, UK, provided *UAS-Dad* (32); L. Partridge, University College London, London, UK, provided *w*^*1118*^; A. McGregor, Oxford Brookes University, Oxford, UK, provided *D. simulans, D. sechellia, D. yakuba, D. pseudoobscura*, and *D. virilis*; and the Gulbenkian Science Institute provided *D. erecta, D. persimilis,* and *D. mojavensis.*

### Fly Husbandry.

Flies were maintained on standard cornmeal agar food (12.5 g of agar, 75 g of cornmeal, 93 g of glucose, 31.5 g of inactivated yeast, 8.6 g of potassium sodium tartrate, 0.7 g of calcium, and 2.5 g of Nipagen [dissolved in 12 mL of ethanol] per liter) at 25 °C on a 12:12-h light:dark cycle. Flies for the proteomics analysis were reared on Lewis medium (65). Males for *SP* knockdown or those with *tub-GAL80*^*ts*^ were shifted to 29 °C on eclosion to activate UAS-transgenes.

### Staining and Immunostaining of Fly Reproductive Tracts.

Unless otherwise stated, 3- to 4-d-old virgin males were used for AG dissection and for mating experiments; 4- to 7-d-old *w*^*1118*^ virgin females were used for mating experiments. For fixed tissues, reproductive tracts were dissected in 4% paraformaldehyde (Sigma-Aldrich) in phosphate-buffered saline (PBS) (Gibco). AGs with the ejaculatory duct attached were fixed for 20 min and rinsed at least four times in PBS prior to further treatments. For females, the abdomen was carefully opened up, and fixative was allowed to permeate internally for 20 min prior to removal of the uterus with seminal receptacle, spermathecae, and common oviduct attached. Reproductive tracts were washed four times with PBS.

Fixed AGs were stained at room temperature in the following solutions and then washed four times in PBS: 1:50 dilution in PBS of a 10 mg/mL solution of Nile red (Sigma-Aldrich) dissolved in acetone and incubated for 30 min; 1:100 dilution in PBS of LysoTracker Deep Red (Life Technologies) for 1 h; 1:50 dilution in PBS of LipidTox (Invitrogen) for 1 h; 1:40 dilution in diluent C of a 1 mM stock of PKH26 red fluorescent cell marker (Sigma-Aldrich) for 30 min; 1:1,000 dilution in PBS of a 10 mg/mL stock of Hoechst 33342 (Invitrogen) for 5 min.

For live imaging, AGs were dissected in ice-cold PBS. Live glands requiring staining were treated for 5 min in a 1:100 dilution of LysoTracker Red DND-99 (Life Technologies) in ice-cold PBS.

For ANCE antibody staining, fixed AGs were permeabilized for 6 × 10 min in PBST (1× PBS, 0.3% Triton X-100 [Sigma-Aldrich]), blocked for 30 min in PBSTG (PBST, 10% goat serum [Sigma-Aldrich]), and incubated overnight at 4 °C in rabbit anti-ANCE primary antibody (66) diluted 1:2,000 in PBSTG. Glands were then washed for 6 × 10 min in PBST before incubation in a 1:400 dilution of Cy-5–conjugated donkey anti-rabbit secondary antibody (The Jackson Laboratory) for 2 h at room temperature. Glands were further washed in PBST for 6 × 10 min prior to mounting.

Glands stained with Hoechst were mounted in PBS; all other fixed reproductive tracts were mounted in Vectashield with DAPI (Vector Laboratories). Glands for live imaging were mounted in a small drop of ice-cold PBS surrounded by 10S Voltalef (VWR) halocarbon oil (67).

### Electron Microscopy.

The 3-d-old *w*^*1118*^ male reproductive tracts were dissected and incubated overnight in 2.5% glutaraldehyde and 4% formaldehyde in PBS (pH 7.2). Glands were then washed with PBS, refixed in 1% osmium tetroxide (Agar Scientific) for 20 min, washed three times in distilled water, and dehydrated through a graded alcohol series and incubated in ethanol and Spurr’s epoxy resin (1:1) (Agar Scientific). Glands were embedded in 100% Spurr’s epoxy resin between two sheets of polythene and polymerized overnight at 60 °C. Ultrathin sections were prepared with a Reichert Ultracut R Ultramicrotome (Leica Biosystems) and mounted on formvar-coated slot grids (Agar Scientific). Sections were contrasted with 2% uranyl acetate and lead citrate (Agar Scientific) and imaged using a JEOL 1010 electron microscope (80 kV).

### Imaging.

Images of fixed reproductive tracts were acquired either on a Zeiss LSM 510 Meta [Axioplan2] or a LSM 880 laser scanning confocal microscope equipped with Zeiss 10× numerical aperture (N.A.) 0.45, 20× N.A. 0.8, 40× N.A. 1.3, and 63× N.A. 1.4 objectives. Live scanning confocal imaging was performed on a Zeiss LSM 710 microscope using a 63× N.A. 1.4 objective. Live DIC images were acquired on a DeltaVision Elite wide-field fluorescence deconvolution microscope (GE Healthcare Life Sciences) equipped with a 100×, N.A. 1.4 UPlanSApo oil objective (Olympus).

### Automated Analysis of Microcarrier Size.

Images were opened with Fiji software. Microcarrier image analysis was performed using the open‐access CellProfiler Software version 2.2.0. A workflow for segmenting all the microcarriers and measuring the minimum Feret diameter of each microcarrier was developed by adding preprogrammed algorithmic modules in a pipeline. Histograms based on microcarrier minimum width and microcarrier area in different minimum width ranges were plotted using GraphPad Prism-8 software.

Changes in microcarrier size were further assessed by recording the presence or absence of microcarriers with a minimum width greater than 10 µm for 7 to 10 glands in a representative 100-µm^2^ field of view of the lumen midway along the length of the gland. *P* values were calculated using Fisher’s exact test.

### Proteomics Analysis.

For the proteomics experiments, we followed the mating, sample preparation methods, liquid chromatography tandem mass spectrometry (LC-MS/MS) analysis, and mass spectrometry (MS) data processing pipeline described by Sepil et al. (8). We detected 2,246 proteins in total but restricted our analysis to the 1,502 detected on the basis of at least two unique peptides (as in refs. 8 and 68). This subset contained 118 SFPs known from previous work to be transferred to females (8, 42, 46, 55). One additional SFP was included that was not previously demonstrated to be transferred to females (intrepid, *intr*), due to its known role within the SP network pathway (42), and three proteins were excluded (*SI Appendix*). All analyses were conducted using R statistical software (version 3.5.1) in RStudio (version 1.1.456). In each analysis, log2 transformed values were used to standardize variance across the dynamic range of protein abundances. Further details of methods and additional data analysis are given in *SI Appendix*.

All other materials, tools, and datasets generated in this study are presented in the paper and *SI Appendix*.

## Supplementary Material

Supplementary File

Supplementary File

## Data Availability

The MS proteomics data have been deposited in the ProteomeXchange Consortium via the PRIDE (69) partner repository with the dataset identifier PXD021897 and DOI: 10.6019/PXD021897. All other study data are included in the article and/or *SI Appendix*.

## References

[1] D. Montagnon, B. Valtat, F. Vignon, M. H. Koll-Back, Secretory proteins of human seminal vesicles and their relationship to lipids and sugars. Andrologia 22 (suppl. 1), 193–205 (1990). 2132070 10.1111/j.1439-0272.1990.tb02085.x

[2] T. L. Veveris-Lowe, S. J. Kruger, T. Walsh, R. A. Gardiner, J. A. Clements, Seminal fluid characterization for male fertility and prostate cancer: Kallikrein-related serine proteases and whole proteome approaches. Semin. Thromb. Hemost. 33, 87–99 (2007). 17253195 10.1055/s-2006-958467

[3] J. Vitku, L. Kolatorova, R. Hampl, Occurrence and reproductive roles of hormones in seminal plasma. Basic Clin. Androl. 27, 19 (2017). 29046808 10.1186/s12610-017-0062-y PMC5640966

[4] G. Pampalakis, G. Sotiropoulou, Tissue kallikrein proteolytic cascade pathways in normal physiology and cancer. Biochim. Biophys. Acta 1776, 22–31 (2007). 17629406 10.1016/j.bbcan.2007.06.001

[5] C. Wilson, A. Leiblich, D. C. Goberdhan, F. Hamdy, The *Drosophila* accessory gland as a model for prostate cancer and other pathologies. Curr. Top. Dev. Biol. 121, 339–375 (2017). 28057306 10.1016/bs.ctdb.2016.06.001 PMC5224695

[6] A. Bairati, Structure and ultrastructure of the male reproductive system in *Drosophila melanogaster*: The genital duct and accessory glands. Monit. Zool. Ital. 2, 105–182 (1968).

[7] L. K. Sirot, Molecular social interactions: *Drosophila melanogaster* seminal fluid proteins as a case study. Adv. Genet. 68, 23–56 (2009). 20109658 10.1016/S0065-2660(09)68002-0 PMC3925388

[8] I. Sepil, Quantitative proteomics identification of seminal fluid proteins in male *Drosophila melanogaster*. Mol. Cell. Proteomics 18, S46–S58 (2019). 30287546 10.1074/mcp.RA118.000831 PMC6427238

[9] P. S. Chen, A male accessory gland peptide that regulates reproductive behavior of female *D. melanogaster*. Cell 54, 291–298 (1988). 3135120 10.1016/0092-8674(88)90192-4

[10] E. Kubli, Sex-peptides: Seminal peptides of the *Drosophila* male. Cell. Mol. Life Sci. 60, 1689–1704 (2003). 14504657 10.1007/s00018-003-3052 PMC11146071

[11] T. Chapman, The sex peptide of *Drosophila melanogaster*: Female post-mating responses analyzed by using RNA interference. Proc. Natl. Acad. Sci. U.S.A. 100, 9923–9928 (2003). 12893873 10.1073/pnas.1631635100 PMC187888

[12] H. Liu, E. Kubli, Sex-peptide is the molecular basis of the sperm effect in *Drosophila melanogaster*. Proc. Natl. Acad. Sci. U.S.A. 100, 9929–9933 (2003). 12897240 10.1073/pnas.1631700100 PMC187889

[13] F. W. Avila, K. Ravi Ram, M. C. Bloch Qazi, M. F. Wolfner, Sex peptide is required for the efficient release of stored sperm in mated *Drosophila* females. Genetics 186, 595–600 (2010). 20679516 10.1534/genetics.110.119735 PMC2954482

[14] C. Ribeiro, B. J. Dickson, Sex peptide receptor and neuronal TOR/S6K signaling modulate nutrient balancing in Drosophila. Curr. Biol. 20, 1000–1005 (2010). 20471268 10.1016/j.cub.2010.03.061

[15] G. B. Carvalho, P. Kapahi, D. J. Anderson, S. Benzer, Allocrine modulation of feeding behavior by the Sex Peptide of *Drosophila*. Curr. Biol. 16, 692–696 (2006). 16581515 10.1016/j.cub.2006.02.064 PMC2745344

[16] P. Cognigni, A. P. Bailey, I. Miguel-Aliaga, Enteric neurons and systemic signals couple nutritional and reproductive status with intestinal homeostasis. Cell Metab. 13, 92–104 (2011). 21195352 10.1016/j.cmet.2010.12.010 PMC3038267

[17] J. Apger-McGlaughon, M. F. Wolfner, Post-mating change in excretion by mated Drosophila melanogaster females is a long-term response that depends on sex peptide and sperm. J. Insect Physiol. 59, 1024–1030 (2013). 23891750 10.1016/j.jinsphys.2013.07.001 PMC3923306

[18] R. E. Isaac, C. Li, A. E. Leedale, A. D. Shirras, *Drosophila* male sex peptide inhibits siesta sleep and promotes locomotor activity in the post-mated female. Proc. Biol. Sci. 277, 65–70 (2010). 19793753 10.1098/rspb.2009.1236 PMC2842620

[19] J. Peng, P. Zipperlen, E. Kubli, *Drosophila* sex-peptide stimulates female innate immune system after mating via the Toll and Imd pathways. Curr. Biol. 15, 1690–1694 (2005). 16169493 10.1016/j.cub.2005.08.048

[20] E. Bath, Sperm and sex peptide stimulate aggression in female Drosophila. Nat. Ecol. Evol. 1, 0154 (2017). 28580431 10.1038/s41559-017-0154 PMC5447820

[21] L. Scheunemann, A. Lampin-Saint-Amaux, J. Schor, T. Preat, A sperm peptide enhances long-term memory in female Drosophila. Sci. Adv. 5, eaax342 (2019). 10.1126/sciadv.aax3432 PMC6867886 31799390

[22] J. Peng, Gradual release of sperm bound sex-peptide controls female postmating behavior in *Drosophila*. Curr. Biol. 15, 207–213 (2005). 15694303 10.1016/j.cub.2005.01.034

[23] E. V. Domanitskaya, H. Liu, S. Chen, E. Kubli, The hydroxyproline motif of male sex peptide elicits the innate immune response in *Drosophila* females. FEBS J. 274, 5659–5668 (2007). 17922838 10.1111/j.1742-4658.2007.06088.x

[24] P. Saudan, Ductus ejaculatorius peptide 99B (DUP99B), a novel *Drosophila melanogaster* sex-peptide pheromone. Eur. J. Biochem. 269, 989–997 (2002). 11846801 10.1046/j.0014-2956.2001.02733.x

[25] M. Tsuda, J. B. Peyre, T. Asano, T. Aigaki, Visualizing molecular functions and cross-species activity of sex-peptide in *Drosophila*. Genetics 200, 1161–1169 (2015). 26022240 10.1534/genetics.115.177550 PMC4574256

[26] N. Yapici, Y. J. Kim, C. Ribeiro, B. J. Dickson, A receptor that mediates the post-mating switch in Drosophila reproductive behaviour. Nature 451, 33–37 (2008). 18066048 10.1038/nature06483

[27] M. Häsemeyer, N. Yapici, U. Heberlein, B. J. Dickson, Sensory neurons in the *Drosophila* genital tract regulate female reproductive behavior. Neuron 61, 511–518 (2009). 19249272 10.1016/j.neuron.2009.01.009

[28] C. H. Yang, Control of the postmating behavioral switch in *Drosophila* females by internal sensory neurons. Neuron 61, 519–526 (2009). 19249273 10.1016/j.neuron.2008.12.021 PMC2748846

[29] Z. Ding, I. Haussmann, M. Ottiger, E. Kubli, Sex-peptides bind to two molecularly different targets in *Drosophila melanogaster* females. J. Neurobiol. 55, 372–384 (2003). 12717705 10.1002/neu.10218

[30] I. U. Haussmann, Y. Hemani, T. Wijesekera, B. Dauwalder, M. Soller, Multiple pathways mediate the sex-peptide-regulated switch in female *Drosophila* reproductive behaviours. Proc. Biol. Sci. 280, 20131938 (2013). 24089336 10.1098/rspb.2013.1938 PMC3790487

[31] B. Zhitomirsky, H. Farber, Y. G. Assaraf, LysoTracker and MitoTracker red are transport substrates of P-glycoprotein: Implications for anticancer drug design evading multidrug resistance. J. Cell. Mol. Med. 22, 2131–2141 (2018). 29377455 10.1111/jcmm.13485 PMC5867146

[32] L. Corrigan, BMP-regulated exosomes from *Drosophila* male reproductive glands reprogram female behavior. J. Cell Biol. 206, 671–688 (2014). 25154396 10.1083/jcb.201401072 PMC4151142

[33] S.-J. Fan, Glutamine deprivation regulates the origin and function of cancer cell exosomes. EMBO J. 39, e103009 (2020). 32720716 10.15252/embj.2019103009 PMC7429491

[34] S. Redhai, Regulation of dense-core granule replenishment by autocrine BMP signalling in *Drosophila* secondary cells. PLoS Genet. 12, e1006366 (2016). 27727275 10.1371/journal.pgen.1006366 PMC5065122

[35] A. Villella, J. B. Peyre, T. Aigaki, J. C. Hall, Defective transfer of seminal-fluid materials during matings of semi-fertile *fruitless* mutants in *Drosophila*. J. Comp. Physiol. A Neuroethol. Sens. Neural Behav. Physiol. 192, 1253–1269 (2006). 16896687 10.1007/s00359-006-0154-1

[36] R. Nolo, L. A. Abbott, H. J. Bellen, *Drosophila Lyra* mutations are gain-of-function mutations of senseless. Genetics 157, 307–315 (2001). 11139511 10.1093/genetics/157.1.307 PMC1461469

[37] A. H. Brand, N. Perrimon, Targeted gene expression as a means of altering cell fates and generating dominant phenotypes. Development 118, 401–415 (1993). 8223268 10.1242/dev.118.2.401

[38] A. H. Brand, A. S. Manoukian, N. Perrimon, Ectopic expression in *Drosophila*. Methods Cell Biol. 44, 635–654 (1994). 7707973 10.1016/s0091-679x(08)60936-x

[39] B. R. Hopkins, BMP signaling inhibition in *Drosophila* secondary cells remodels the seminal proteome and self and rival ejaculate functions. Proc. Natl. Acad. Sci. U.S.A. 116, 24719–24728 (2019). 31740617 10.1073/pnas.1914491116 PMC6900634

[40] K. R. Ram, M. F. Wolfner, Sustained post-mating response in *Drosophila melanogaster* requires multiple seminal fluid proteins. PLoS Genet. 3, e238 (2007). 18085830 10.1371/journal.pgen.0030238 PMC2134937

[41] K. R. Ram, M. F. Wolfner, A network of interactions among seminal proteins underlies the long-term postmating response in Drosophila. Proc. Natl. Acad. Sci. U.S.A. 106, 15384–15389 (2009). 19706411 10.1073/pnas.0902923106 PMC2741260

[42] G. D. Findlay, Evolutionary rate covariation identifies new members of a protein network required for *Drosophila melanogaster* female post-mating responses. PLoS Genet. 10, e1004108 (2014). 24453993 10.1371/journal.pgen.1004108 PMC3894160

[43] A. Singh, Long-term interaction between Drosophila sperm and sex peptide is mediated by other seminal proteins that bind only transiently to sperm. Insect Biochem. Mol. Biol. 102, 43–51 (2018). 30217614 10.1016/j.ibmb.2018.09.004 PMC6249070

[44] J. L. Mueller, Cross-species comparison of *Drosophila* male accessory gland protein genes. Genetics 171, 131–143 (2005). 15944345 10.1534/genetics.105.043844 PMC1456506

[45] V. R. Chintapalli, J. Wang, J. A. Dow, Using FlyAtlas to identify better *Drosophila melanogaster* models of human disease. Nat. Genet. 39, 715–720 (2007). 17534367 10.1038/ng2049

[46] G. D. Findlay, X. Yi, M. J. Maccoss, W. J. Swanson, Proteomics reveals novel *Drosophila* seminal fluid proteins transferred at mating. PLoS Biol. 6, e178 (2008). 18666829 10.1371/journal.pbio.0060178 PMC2486302

[47] D. A. Carver, B. A. Ball, Lipase activity in stallion seminal plasma and the effect of lipase on stallion spermatozoa during storage at 5 degrees C. Theriogenology 58, 1587–1595 (2002). 12374128 10.1016/s0093-691x(02)01049-x

[48] B. Sias, Cloning and seasonal secretion of the pancreatic lipase-related protein 2 present in goat seminal plasma. Biochim. Biophys. Acta 1686, 169–180 (2005). 15629686 10.1016/j.bbalip.2004.09.008

[49] L. Anel-López, Analysis of seminal plasma from brown bear (*Ursus arctos*) during the breeding season: Its relationship with testosterone levels. PLoS One 12, e0181776 (2017). 28771486 10.1371/journal.pone.0181776 PMC5542667

[50] F. Vignon, A. Clavert, M. H. Koll-Back, P. Reville, On the glandular origin of seminal plasma lipids in man. Andrologia 24, 341–343 (1992). 1443676 10.1111/j.1439-0272.1992.tb02666.x

[51] N. S. Juyena, J. Vencato, G. Pasini, I. Vazzana, C. Stelletta, Alpaca semen quality in relation to different diets. Reprod. Fertil. Dev. 25, 683–690 (2013). 22951252 10.1071/RD12050

[52] Z. Li, Lipid droplets control the maternal histone supply of *Drosophila* embryos. Curr. Biol. 22, 2104–2113 (2012). 23084995 10.1016/j.cub.2012.09.018 PMC3513403

[53] J. A. Olzmann, P. Carvalho, Dynamics and functions of lipid droplets. Nat. Rev. Mol. Cell Biol. 20, 137–155 (2019). 30523332 10.1038/s41580-018-0085-z PMC6746329

[54] L. K. Sirot, M. F. Wolfner, S. Wigby, Protein-specific manipulation of ejaculate composition in response to female mating status in *Drosophila melanogaster*. Proc. Natl. Acad. Sci. U.S.A. 108, 9922–9926 (2011). 21628597 10.1073/pnas.1100905108 PMC3116428

[55] B. R. Hopkins, Divergent allocation of sperm and the seminal proteome along a competition gradient in *Drosophila melanogaster*. Proc. Natl. Acad. Sci. U.S.A. 116, 17925–17933 (2019). 31431535 10.1073/pnas.1906149116 PMC6731677

[56] T. A. Markow, Evolution of *Drosophila* mating systems. Evol. Biol. 29, 73–106 (1996).

[57] M. K. McGeary, G. D. Findlay, Molecular evolution of the sex peptide network in *Drosophila*. J. Evol. Biol. 33, 629–641 (2020). 31991034 10.1111/jeb.13597

[58] Y. Shiga, M. Tanaka-Matakatsu, S. Hayashi, A nuclear GFP/B-galactosidase fusion protein as a marker for morphogenesis in living *Drosophila*. Dev. Growth Differ. 38, 99–106 (1996).

[59] T. Lee, L. Luo, Mosaic analysis with a repressible cell marker for studies of gene function in neuronal morphogenesis. Neuron 22, 451–461 (1999). 10197526 10.1016/s0896-6273(00)80701-1

[60] L. A. Perkins, The transgenic RNAi project at Harvard Medical school: Resources and validation. Genetics 201, 843–852 (2015). 26320097 10.1534/genetics.115.180208 PMC4649654

[61] S. E. McGuire, P. T. Le, A. J. Osborn, K. Matsumoto, R. L. Davis, Spatiotemporal rescue of memory dysfunction in *Drosophila*. Science 302, 1765–1768 (2003). 14657498 10.1126/science.1089035

[62] S. Hayashi, GETDB, a database compiling expression patterns and molecular locations of a collection of Gal4 enhancer traps. Genesis 34, 58–61 (2002). 12324948 10.1002/gene.10137

[63] E. J. Rideout, A. J. Dornan, M. C. Neville, S. Eadie, S. F. Goodwin, Control of sexual differentiation and behavior by the doublesex gene in Drosophila melanogaster. Nat. Neurosci. 13, 458–466 (2010). 20305646 10.1038/nn.2515 PMC3092424

[64] V. Greco, M. Hannus, S. Eaton, Argosomes: A potential vehicle for the spread of morphogens through epithelia. Cell 106, 633–645 (2001). 11551510 10.1016/s0092-8674(01)00484-6

[65] E. B. Lewis, A new standard food medium. Drosophila Information Service 34, 117–118 (1960).

[66] C. M. Rylett, M. J. Walker, G. J. Howell, A. D. Shirras, R. E. Isaac, Male accessory glands of *Drosophila melanogaster* make a secreted angiotensin I-converting enzyme (ANCE), suggesting a role for the peptide-processing enzyme in seminal fluid. J. Exp. Biol. 210, 3601–3606 (2007). 17921161 10.1242/jeb.009035

[67] R. M. Parton, A. M. Vallés, I. M. Dobbie, I. Davis, Live cell imaging in *Drosophila melanogaster*. Cold Spring Harb. Protoc. 2010, pdb.top75 (2010). 20360379 10.1101/pdb.top75

[68] K. Borziak, A. Álvarez-Fernández, T. L Karr, T. Pizzari, S. Dorus, The Seminal fluid proteome of the polyandrous Red junglefowl offers insights into the molecular basis of fertility, reproductive ageing and domestication. Sci. Rep. 6, 35864 (2016). 27804984 10.1038/srep35864 PMC5090203

[69] Y. Perez-Riverol, The PRIDE database and related tools and resources in 2019: Improving support for quantification data. Nucleic Acids Res. 47, D442–D450 (2019). 30395289 10.1093/nar/gky1106 PMC6323896

