# Supplementary material for: Drosophila Sex Peptide controls the assembly of lipid microcarriers in seminal fluid
Source: Proc Natl Acad Sci U S A. 2021 Jan 25;118(5):e2019622118. doi: 10.1073/pnas.2019622118 (PMC7865141; doi:10.1073/pnas.2019622118)
Supplement: Supplementary File [file pnas.2019622118.sapp.pdf]

Supplementary Information for:

Drosophila Sex Peptide Controls the Assembly of Lipid Microcarriers in Seminal Fluid

S. Mark Wainwright[a], Ben R. Hopkins[b,c], Cláudia C. Mendes[a], Aashika Sekar[a], Benjamin Kroeger[a], Josephine E.E.U. Hellberg[a], Shih-Jung Fan[a], Abigail Pavey[a], Pauline P. Marie[a], Aaron Leiblich[a], Irem Sepil[b], Philip D. Charles[d], Marie L. Thézénas[d], Roman Fischer[d], Benedikt M. Kessler[d], Carina Gandy[a], Laura Corrigan[a], Rachel Patel[a], Stuart Wigby[b,e,f], John F. Morris[a], Deborah C.I. Goberdhan[a], Clive Wilson[a]

[a] Department of Physiology, Anatomy and Genetics, University of Oxford, South Parks Road, Oxford, OX1 3QX, UK

[b] Department of Zoology, University of Oxford, Oxford, OX1 3PS, UK

[c] Department of Evolution and Ecology, University of California, Davis, CA 95616, USA

[d] Target Discovery Institute (TDI) Mass Spectrometry Laboratory, Target Discovery Institute, Nuffield Department of Medicine, University of Oxford, Oxford, OX3 7BN, UK

[e] Applied Zoology, Faculty Biology, Technische Universität Dresden, Dresden, D-01069, Germany

[f] Institute of Infection, Veterinary and Ecological Sciences, University of Liverpool, L69 7ZB Liverpool, United Kingdom

E-mail: clive.wilson@dpag.ox.ac.uk

This PDF file includes:

Supplementary Materials and Methods

Figures S1 to S6

Legend for SI Movie 1

SI Appendix

Supplementary Materials and Methods

Proteomics analysis

A single sample consisted of 17 pairs of accessory glands, which was dissected on ice under a light microscope in a drop of ice-cold PBS. Grouping of accessory glands enhanced ability to detect otherwise low abundance proteins (1). The accessory glands were severed from the distal end of the ejaculatory duct and the seminal vesicles and testes removed. Samples were transferred to 25 µl of PBS and stored at -80°C prior to processing. In total, 12 samples were generated: fully factorial permutations of mated status (virgin/mated) and genotype (*SP* null/control) across 3 replicates.

To prepare the samples for proteomics analysis, the Gel-Aided Sample Preparation (GASP) protocol (2) was used, which we have previously shown achieves highly-sensitive detection of SFPs (1). The glandular tissue was macerated using a pestle for one minute. 25µl of RIPA (radioimmunoprecipitation assay) buffer (Pierce) was added to lyse the cells, dripping it slowly over the pestle to wash residual tissue back into the sample. The lysate was then incubated on ice with 50 mM of dithiothreitol. An equal volume of 40% acrylamide/bisacrylamide solution (37.5:1; National Diagnostics), 5µl of 10% ammonium persulphate, and 5 µl of tetramethylethylenediamine were added in succession. This led to the formation of a solid gel, which was loaded on to a spin shredder (CLS9301, Sigma/Corning) and fragmented. The resulting pieces of gel were fixed in a solution of 5% acetic acid/40% ethanol before undertaking two rounds of alternating thiourea/urea and acetonitrile (ACN) washes, followed by a further two rounds of ammonium bicarbonate and ACN washes. Then

250µl of dilute trypsin was added and the solution incubated at 37°C overnight. The resulting peptides were extracted via two repeated ACN replacements before drying and desalting them in Sola SPE columns (Thermo Scientific). Prior to LC-MS/MS, the peptides were suspended in a 2% ACN/0.1% formic acid buffer.

For peptide analysis, we used a LC-MS/MS platform composed of a Dionex Ultimate 3000 and a Q-Exactive HF mass spectrometer (Thermo Scientific). Peptide loading took place in a solution of 0.1% TFA in 2% ACN on a trap column (PepMAP C18, 300µm x 5m, 5µm particle; Thermo Scientific). Peptides were separated using an easy spray column (PepMAP C18, 75 µm x 500 mm, 2 µm particle size; Thermo Scientific) with a gradient of 2% ACN to 35% ACN in 0.1% FA in 5% DMSO. For MS spectra collection, a resolution of 60,000 was used in profile mode on the Q-Exactive HF (ion target = 3x106). The top 12 most intense features were selected for subsequent MS/MS analysis (resolution of 30,000). The following parameters were set: dynamic exclusion = 27 seconds; AGC target = 1x105; isolation width = 1.2 m/z; and maximum acquisition time = 45ms.

MS data processing

The MS data processing pipeline employed has previously been described (1). RAW data were imported into Progenesis QIP (version 4.1.6675.48614), exporting spectra as MGF files using the 200 most intense peaks without deconvolution for searching. For peptide identification, the *Drosophila melanogaster* UniProt reference proteome was used as a search target, with database retrieval conducted on 30/03/2015 (21361 sequences) in Mascot 2.5.1. The search parameters incorporated the following: Oxidation (M) as a variable modifications;

Propionamide (K), Propionamide (N-term) and Propionamide (C) as fixed modifications; one missed cleavage sites; 0.05 Da fragment mass accuracy; 10 ppm precursor accuracy. Prior to importing the search results into Progenesis for quantification via the Top3 method, a peptide-level 1% FDR was applied alongside a further Mascot ion cut-off of 20. The resulting protein abundance data were subsequently normalised using the internal Progenesis algorithm to a set of housekeeping proteins.

Data analysis

As expected, significantly lower abundance of SP was detected in the *SP* null treatment compared to controls (LM: $F_{1,12}$=30.726, p=0.0009). However, surprisingly SP peptides were detected in all null male samples at approximately 17% of control abundance, which we attribute to read-through of the stop codon in the null allele, a common event in *Drosophila* (3). Whether this protein is correctly processed and secreted remains unclear. To confirm that the presence of SP in our null samples was not a result of carry-over from previous control samples run on the MS, we independently re-ran two *SP* null samples: again, SP was detected, thus verifying our findings. SP was omitted from further analysis to avoid the confound of attributing changes to the SFP proteome to altered SP levels. We further omitted S-Lap7 and Spn28Db due to high between-replicate variability.

Hierarchical clustering analyses were performed on $\log_2$ abundances using a Pearson correlation distance metric and graphically displayed using the *pheatmap* package. For each protein, a mean abundance was taken across replicates. The first five earliest-branching clusters were selected for further analysis. To visualise the general abundance profile across

treatments that each cluster captured, the non-$\log_2$ transformed abundance for each protein was divided by the mean calculated across all samples and averaged across replicates. Mean centring in this way gives a measure of abundance change that is comparable across the substantial variance range of proteins. We tested the significance of a relationship between the protein abundances within a cluster and our measured variables using linear mixed effects models that modelled protein identity as a random effect and mating and genotype as fixed effects. Protein abundances were averaged across the three replicates for each treatment combination to improve the model fit, which was inferred through visual inspection of diagnostic plots. The statistical significance of factors was assessed by analysis of deviance using the 'drop1' function. Where the interaction term was insignificant, the model was re-fitted without it.

To identify proteins that are not classified as SFPs, but which have a profile that suggests they might fail to be transferred or be transferred in excess quantities in *SP* null versus rescue males, we iterated a linear model over every non-SFP protein in our dataset. A hierarchical clustering analysis (as above) was then performed on all proteins that gave a significant ($p<0.05$) interaction between genotype and mating status.

PCAs were performed using the 'prncomp' function in the *stats* package and produced using *ggbiplot*.

References for SI appendix

1. I. Sepil *et al.*, Quantitative proteomics identification of seminal fluid proteins in male *Drosophila melanogaster. Mol. Cell Proteomics* **18,** S46-58 (2019).

2. R. Fischer, B. M. Kessler. Gel-aided sample preparation (GASP) - A simplified method for gel-assisted proteomic sample generation from protein extracts and intact cells. *Proteomics* **15**, 1224–1229 (2015).

3. J. G. Dunn, C. K. Foo, N. G. Belletier, E. R. Gavis, J. S. Weissman (2013). Ribosome profiling reveals pervasive and regulated stop codon readthrough in Drosophila melanogaster. *ELife*, **2**, e01179.

Supplementary Figures.

Fig. S1. Structure and cargos of accessory gland microcarriers.

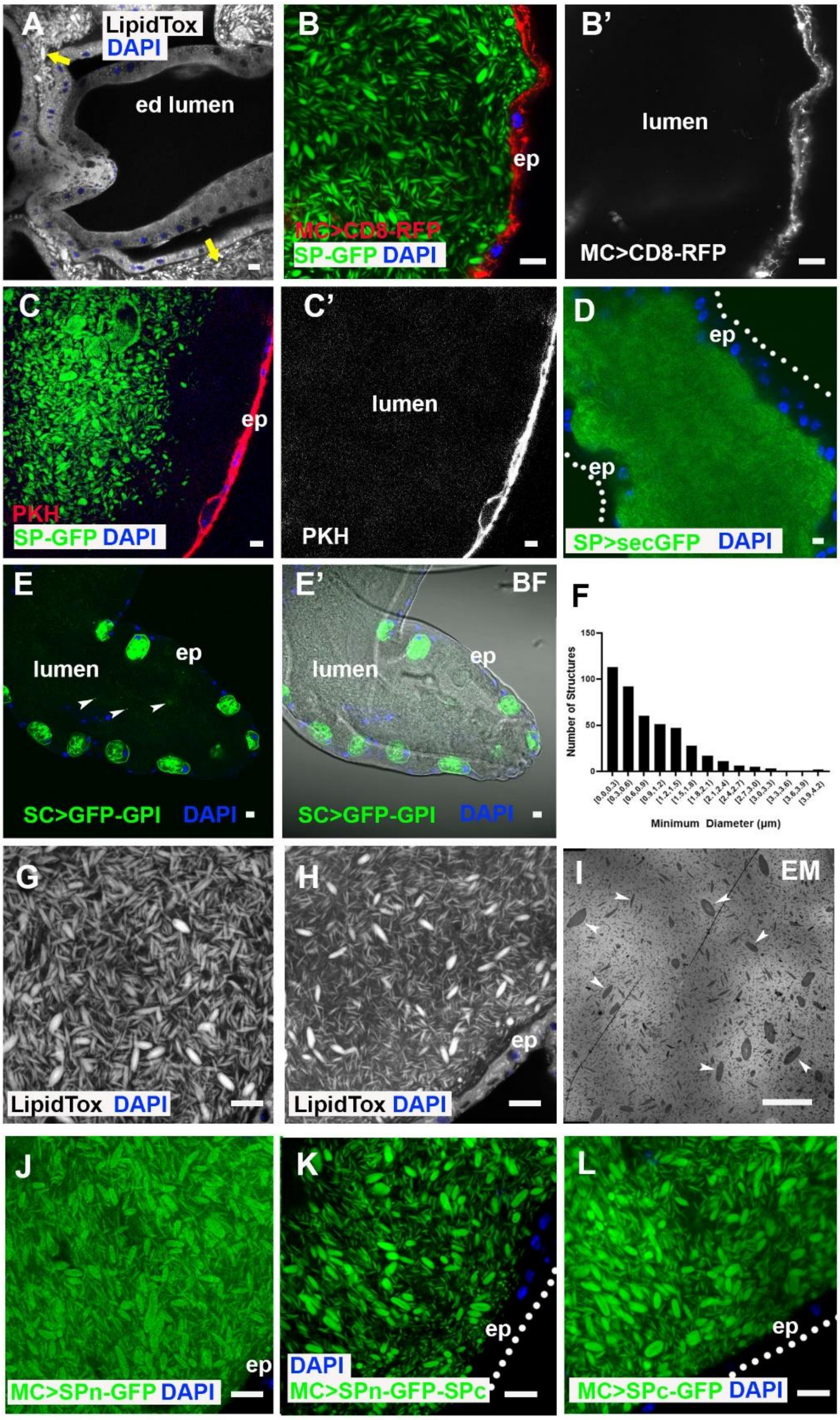

Fig. S1. Structure and cargos of accessory gland microcarriers.

(A) LipidTox staining of male reproductive tract, showing ejaculatory duct (ed) lumen in centre and the two lobes of the accessory gland at the top and bottom, which contain microcarriers (yellow arrows), unlike the ejaculatory duct lumen. (B, C) Both main cell-expressed transmembrane CD8-RFP (B) and the lipid bilayer dye PKH26 (C) mark the main cell apical membrane (B', C'), but not the luminal microcarriers labelled by SP-GFP (merge in B, C). (D) Secreted GFP is not preferentially partitioned on to microcarriers. (E) In the AG lumen, secreted secondary cell-expressed GFP-GPI is primarily associated with puncta (arrowheads; cell boundaries seen in bright-field image; E') and does not appear to be loaded on to microcarriers. (F) Size distribution of microcarriers in LipidTox-stained AG lumen from image in Fig. 1B'. (G, H) Microcarriers still form in accessory glands in which secretion of secondary cells has been suppressed by cell-type-specific expression of the BMP signalling antagonist Dad (H). However, microcarrier size generally appears more bimodal than controls (G), with more thin, thread-like structures present. (I) Transmission electron micrograph of AG lumen showing a range of sizes of microcarriers (arrowheads) (J-L). The N-terminus of mature SP with a C-terminal GFP tag (J; SPn-GFP), full length SP with a central GFP tag (K; SPn-GFP-SPc) and the C-terminus of SP with an N-terminal GFP tag (L; GFP-SPc) all concentrate on microcarriers when expressed in main cells albeit at lower levels for SPn-GFP. Nuclei marked with DAPI (blue). AG epithelium (ep). Scale bars, 10 µm.

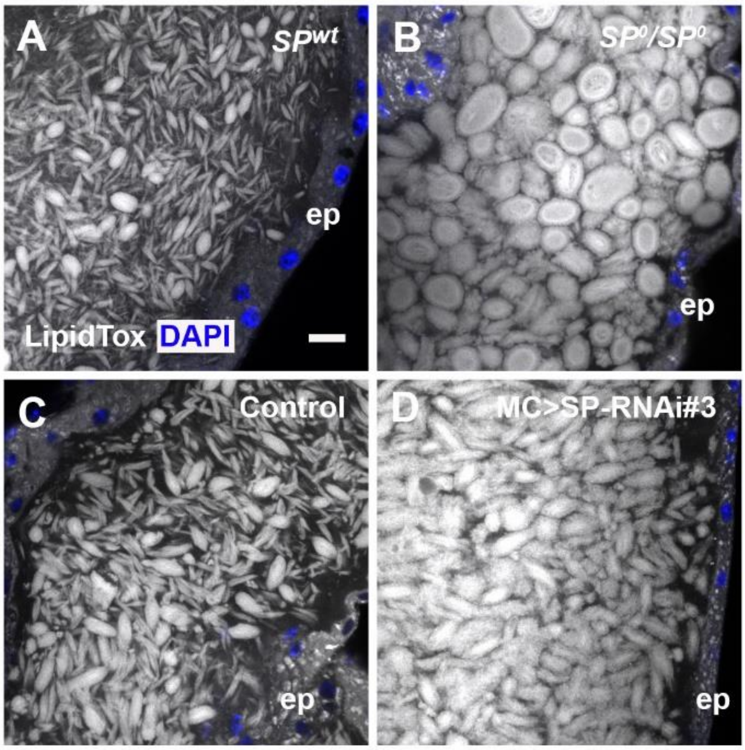

Fig. S2. Loss or reduction of SP disrupts microcarrier morphology.

(A-D) LipidTox-stained microcarriers are highly enlarged in *SP^0/SP^0* homozygous males (B) when compared to controls (A). Expressing a third RNAi targeting *SP* transcripts *(UAS-SP-RNAi#3*; v109175) induces the formation of enlarged microcarriers (D), unlike controls (C). Nuclei marked with DAPI (blue). AG epithelium (ep). Scale bars, 10 µm.

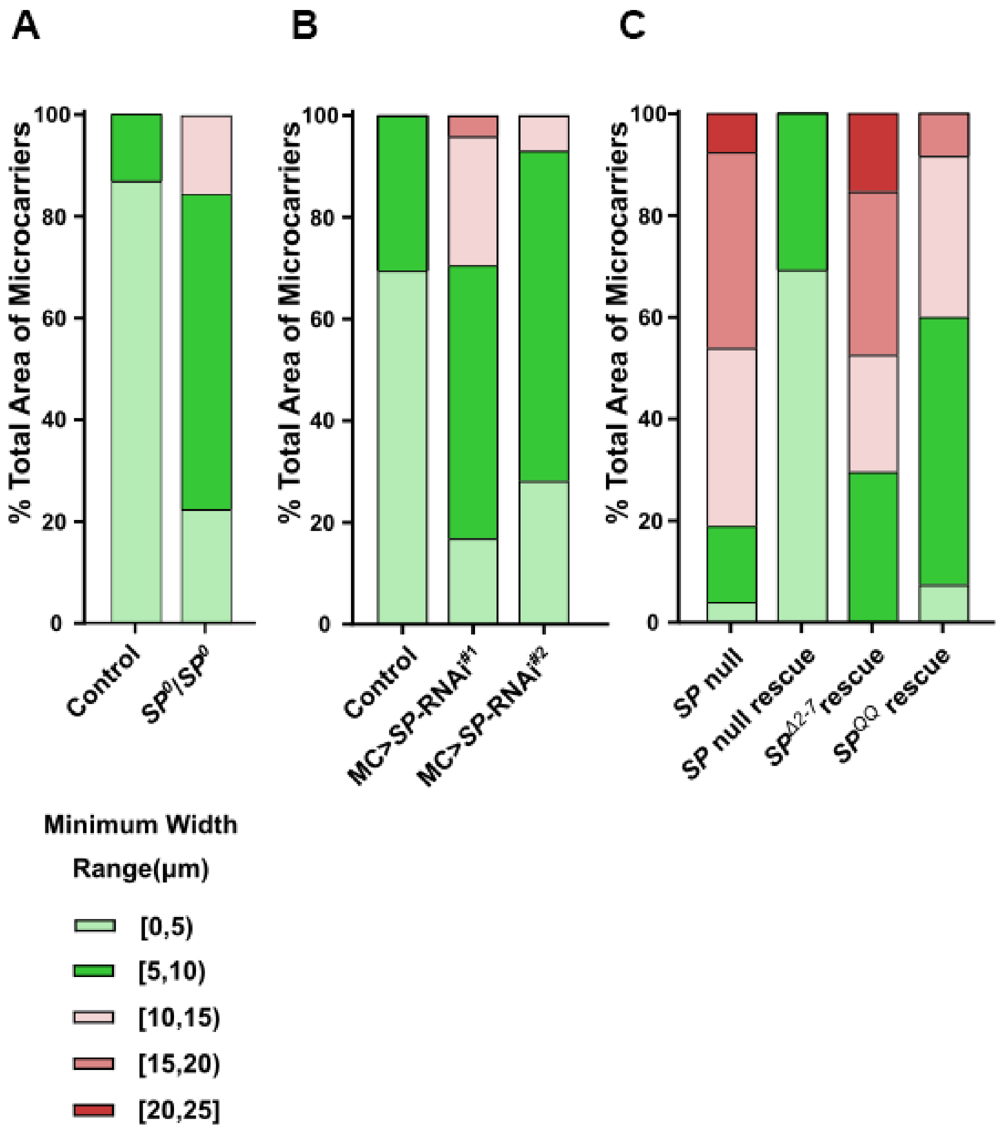

Fig. S3. ***SP*** **mutant,** ***SP*** **RNAi and some** ***SP*** **rescue glands have enlarged microcarriers.**

(A-C) Histograms showing percentage of microcarrier area within given microcarrier minimum width ranges. (A) More lipid is incorporated into microcarriers in larger size ranges in homozygous *SP⁰/SP⁰* gland (from Fig. S2B) than in control glands (Fig. S2A). (B) *SP*-RNAi knockdown in main cells increases the incorporation of lipid into larger microcarriers (from glands shown in Fig. 4B, C) compared with control glands (Fig. 4A). (C) Expression of $SP^{\Delta2\text{-}7}$ or SP^{QQ} (from glands shown in Fig. 4J, K) fails to rescue the $SP^0/Df(SP)$ null phenotype (Fig 4H) when compared with SP^0 $SP^+/Df(SP)$ rescue (Fig. 4I). Main cell (MC).

Figure S4. Loss of *SP* is associated with compositional change in the seminal and 'non-SFP' proteome

A

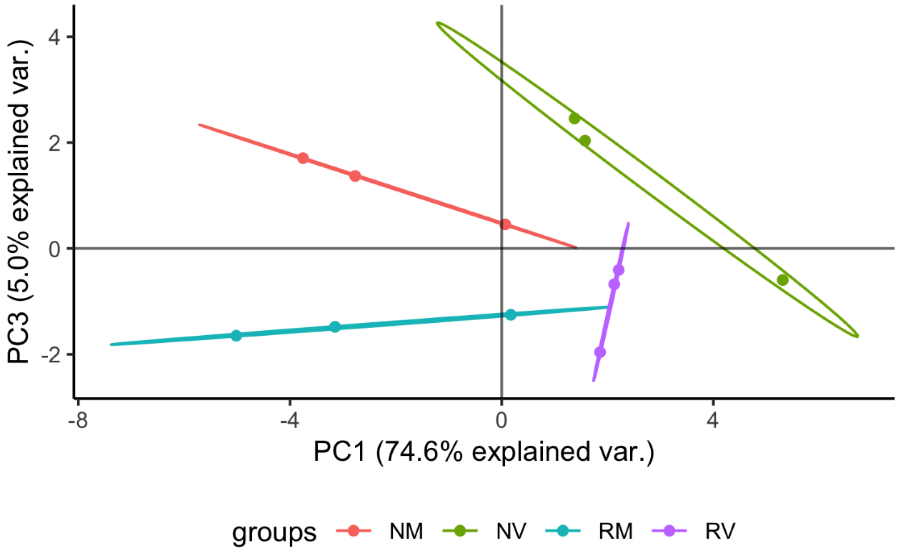

B

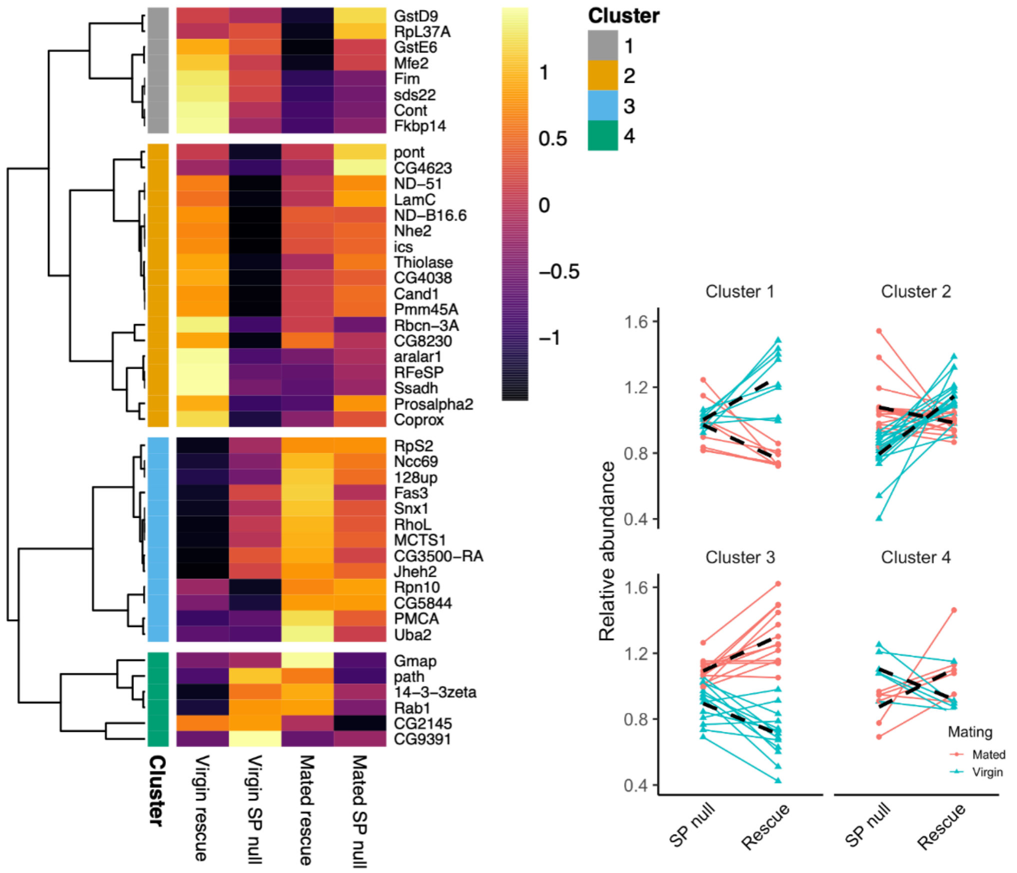

Figure S4. Loss of *SP* is associated with compositional change in the seminal and 'non-SFP' proteome

(A) A biplot of PC1 against PC3 reporting the output of a PCA conducted on seminal fluid proteins (SFPs). PC2 did not show a clear association with genotype. *NM* = *SP* null*,* mated; *NV* = *SP* null*,* virgin; *RM* = Rescue*,* mated; *RV* = Rescue*,* virgin. Ellipses denote 80% probability. (B) Heatmap shows mean log_2 abundance taken across three replicates for each non-SFP protein that showed a significant interaction between mating and genotype. Each protein is plotted for the rescue control virgin (far-left) and mated (middle-right column), and *SP* null virgin (middle-left) and mated (far-right) glands. Mean-centred abundance patterns for each protein in the top 4 highest-order clusters are plotted to the right. N= *SP* null; R= rescue control; red = mated glands; blue = virgin glands. Black dashed lines give the average response for a mating treatment.

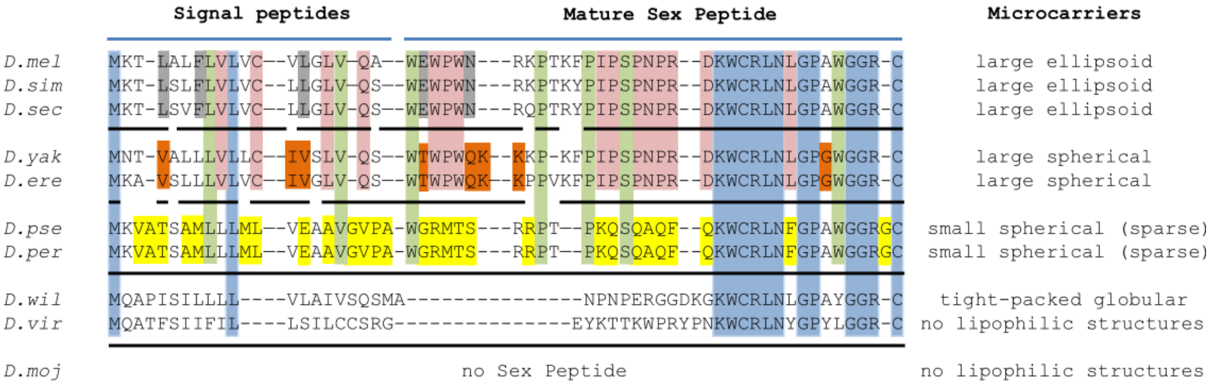

Fig. S5. Rapid evolution of SP sequences correlates with changes in microcarrier morphology, size and abundance in diverse *Drosophila* species.

SP protein sequence alignment for the different *Drosophila* species used in this study. Species are clustered according to subgroup. Conserved amino acids are highlighted or underlined; blue = conserved across all SP-expressing species; green = conserved in *melanogaster* and *obscura* groups; pink = conserved in *melanogaster* group; grey, brown or yellow = conserved within a single species cluster. The subdivision of these groups based on SP protein sequence, correlates with shape, size and abundance of microcarriers in each species. Adapted from (25).

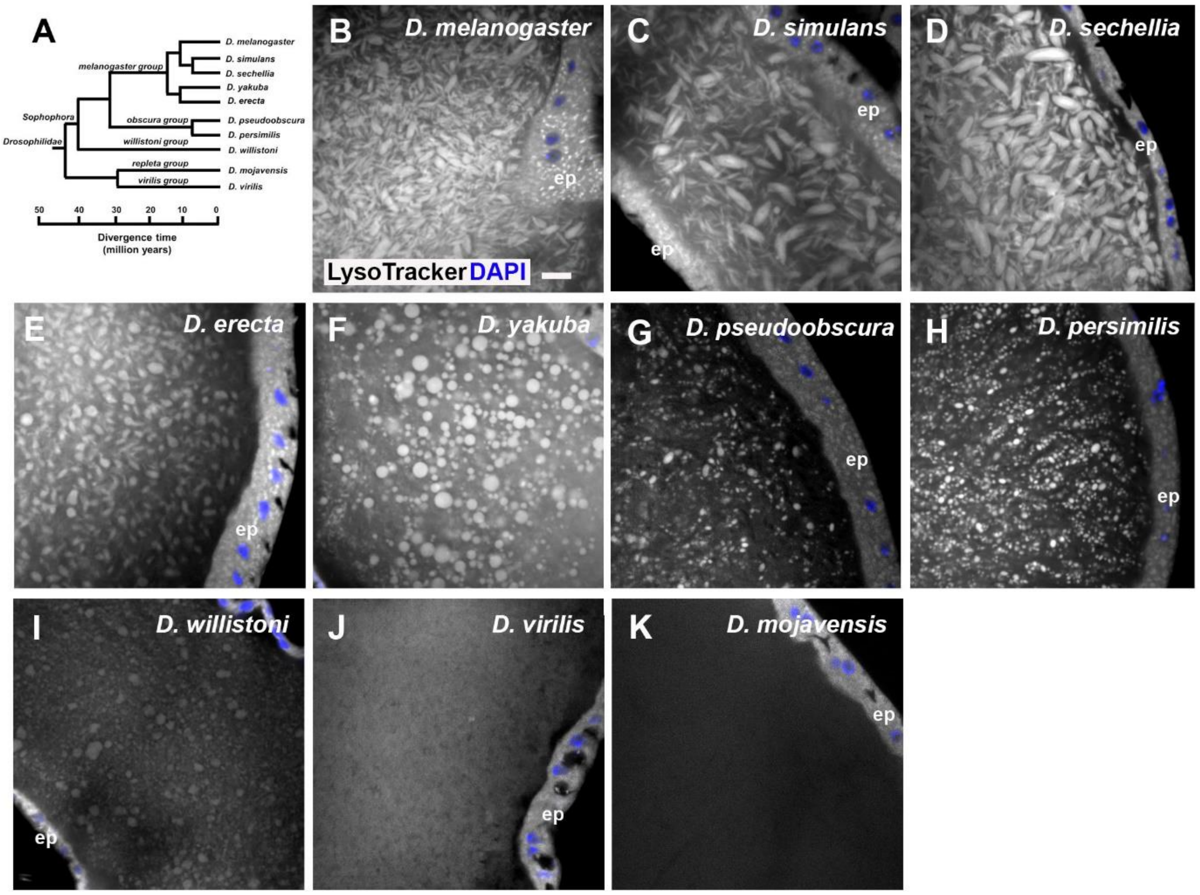

Fig. S6. Co-evolution of microcarrier morphology and SP in *Drosophila* species.

(A) Phylogenetic tree of *Drosophila* species used in this study. All species except *D. mojavensis* have a putative SP homologue. Adapted from http://flybase.org/blast/. (B-K) LysoTracker Deep Red staining of AGs from 6-day-old virgin males from selected *Drosophila* species, namely *D. melanogaster* (B), *D. simulans* (C), *D. sechellia* (D), *D. erecta* (E), *D. yakuba* (F), *D. pseudoobscura* (G), *D. persimilis* (*H*), *D. willistoni* (*I*), *D. virilis* (*J*) and *D. mojavensis* (K). Note that different subgroups have noticeably different microcarrier size, shape and density, and staining correlates with LipidTox staining in Fig. 7. Nuclei marked with DAPI (blue). AG epithelium (ep). Scale bar, 10 µm, applies to all images.

Supplementary Movies

SI Movie 1. Live imaging of SP-GFP-labelled microcarriers.

Z stack through distal tip of live SP-GFP AG. LysoTracker-Red (red; used at high concentrations) marks epithelial layer of gland. Confocal sections were captured at 1.5 µm intervals on *Z* axis.
